# Pyrene-modified PNAs: Stacking interactions and selective excimer emission in PNA_2_DNA triplexes

**DOI:** 10.3762/bjoc.10.154

**Published:** 2014-07-02

**Authors:** Alex Manicardi, Lucia Guidi, Alice Ghidini, Roberto Corradini

**Affiliations:** 1 Department of Chemistry, University of Parma, Parco Area delle Scienze 17/A, 43124, Parma, Italy. Fax: +39 0521 905472; Tel: +39 0521 905410; 2 Present Address: Department of Biosciences and Nutrition, Karolinska Institutet, Novum, Hälsovägen 7, 14183, Huddinge, Sweden

**Keywords:** modified nucleobase, nucleic acids, PNA, pyrene excimer, SNP recognition, triplex stabilization

## Abstract

Pyrene derivatives can be incorporated into nucleic acid analogs in order to obtain switchable probes or supramolecular architectures. In this paper, peptide nucleic acids (PNAs) containing 1 to 3 1-pyreneacetic acid units (**PNA1**–**6**) with a sequence with prevalence of pyrimidine bases, complementary to cystic fibrosis W1282X point mutation were synthesized. These compounds showed sequence-selective switch-on of pyrene excimer emission in the presence of target DNA, due to PNA_2_DNA triplex formation, with stability depending on the number and positioning of the pyrene units along the chain. An increase in triplex stability and a very high mismatch-selectivity, derived from combined stacking and base-pairing interactions, were found for **PNA2**, bearing two distant pyrene units.

## Introduction

**Figure 1:**
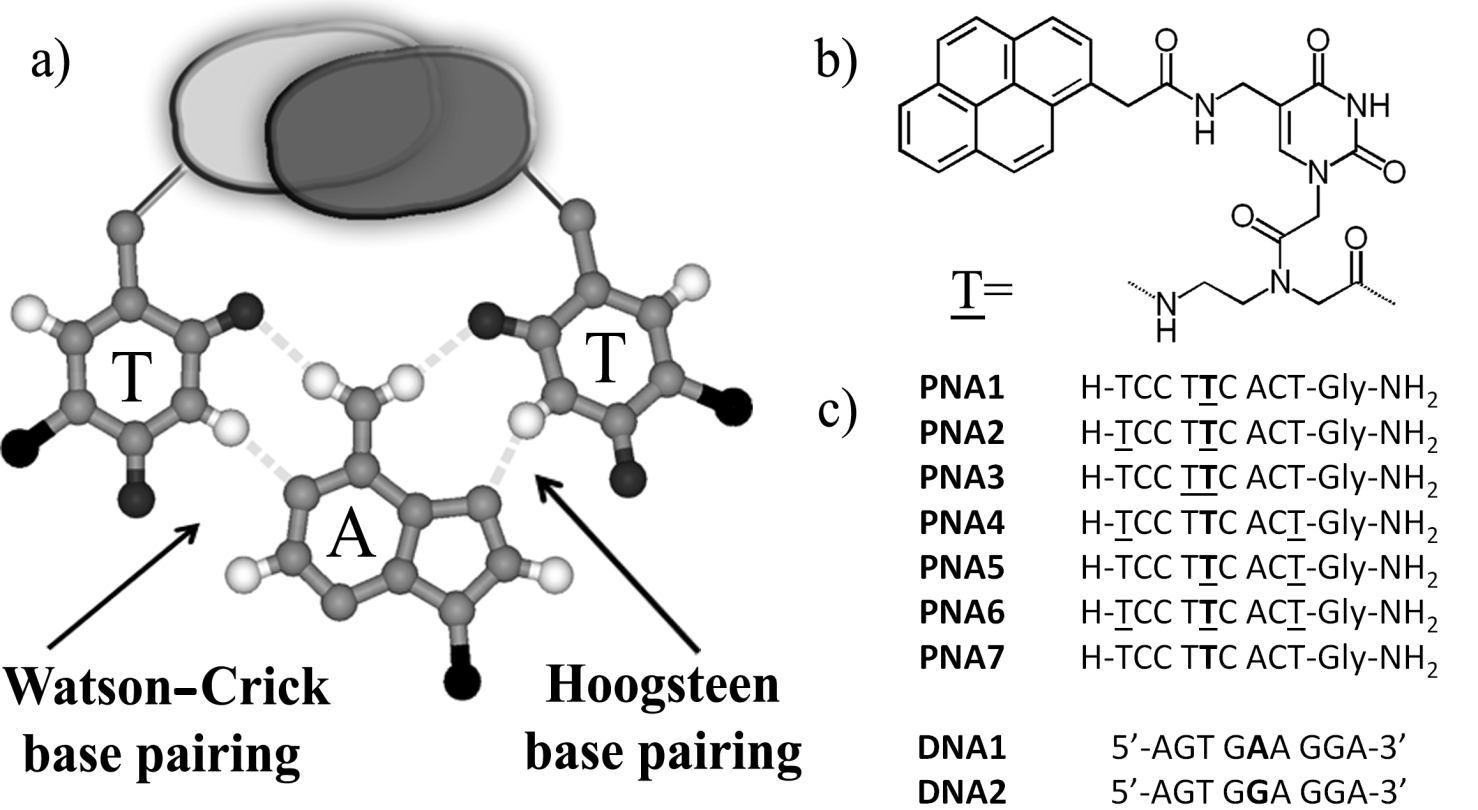
(a) TAT triplet structure showing Watson–Crick and Hoogsteen base pairing; the binding can be reinforced by the concurrent interaction between two groups protruding from C5 position of thymine into the major groove; (b) pyrene-modified uracil derivative used in PNA monomer in the present study; (c) sequences of PNA and DNA used. T indicates pyrene modified nucleobases; bold letters indicate the position of W1282X point mutation.

Peptide nucleic acid (PNA) probes are very selective in the recognition of DNA and have been used in a large variety of diagnostic methods, easily allowing the detection of point mutations at very low concentrations [1–3]. Poly-pyrimidine PNA can form very stable triplexes of the type PNA/DNA/PNA with poly-purine DNA, via both Watson–Crick and Hoogsteen base pairing (Figure 1). These structures are so stable that dsDNA undergoes displacement of the non-complementary strand [4–7]. However, the formation of triplex structures is limited to homopyrimidine sequences since the presence of one or more purine residues destabilizes these complexes and favour the formation of less stable duplexes [8]. Therefore it would be of great value to adopt strategies for the stabilization of triplex structures even in the presence of non-pyrimidine bases. From the available structural data on these complexes [7], it is possible to envisage that any pair of groups protruding from both thymines methyl groups of a TAT triplet and able to give rise to attractive interactions (Figure 1a) would stabilize the triplex. If these groups are aromatic fluorophores, changes in the fluorescence properties can be observed upon interaction with DNA, thus enabling to study the occurring interactions and to produce switching PNA probes.

Fluorescent switching probes for DNA detection are very useful tools in diagnostics applications such as real-time PCR and in situ hybridisation [9–10]. Among the possible reporter groups, pyrene has been proposed in several in vitro detection systems, due to the sensitivity of its fluorescence properties to microenvironment and due to its ability to produce stabilizing stacking interactions and to show excimer fluorescence [11–20]. Furthermore, pyrene has been shown to favour self-assembly processes of supramolecular structures [21–28] and interact with carbon nanostructures such as nanotubes [29] or graphene [30], thus allowing to create composite material with special properties. PNA fluorescent probes bearing pyrene units as “universal base” were described [31–32], and recently, pyrrolidinyl-PNA bearing a uracil-linker pyrene unit showed good fluorescence response and mismatch recognition [33]; though terminal pyrene units were shown to stabilize triplexes formed by oligonucleotide probes [34], the effect of single- or multiple pyrene units on PNA in the formation of triplex structures has still to be addressed.

We have recently reported the modification of uracil at C5 by hydroxymethylation, followed by substitution with chloride and then with azide, which can be used for click chemistry or as a masked amino group both in a PNA monomer and in PNA oligomers, allowing to produce a variety of modified PNAs from a single precursor [35]. This chemistry introduces a moderate degree of flexibility which can be useful for allowing interactions with other groups to occur within the major groove.

In this work we applied this strategy to the synthesis of new mono-, di- and tri-functionalised PNA containing a 1-pyreneacetic acid residue linked to this C5-aminomethyl group (Figure 1b). As a model sequence, we chose a 9-mer (Figure 1c) complementary to a purine-rich tract of DNA which is present in the mutated form of the human cystic fibrosis (CFTR) gene, and which was previously studied in our lab using PNA and modified PNA probes [36–37]; this mutation is characterised by the presence of an adenine instead of guanine, and corresponds to one of the most frequent point mutations connected with cystic fibrosis (M-W1282X).

## Results and Discussion

### Synthesis of the PNA strands

**Scheme 1:**
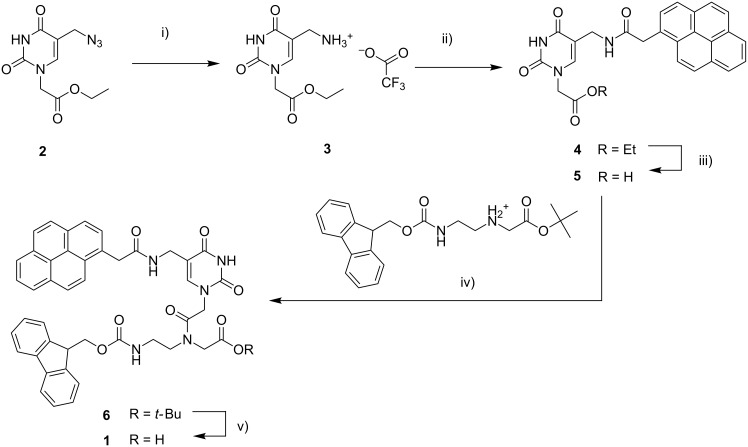
Synthesis of the PNA monomer 1: i) 1. PPh_3_, H_2_O, THF; 2. TFA, 71%; ii) 1-pyreneacetic acid, HBTU, DIPEA, DMF, 66%; iii) NaOH, H_2_O/MeOH (2:1), 91%; iv) EDC·HCl, DIPEA, DhBtOH, DMF, 68%; v) TFA, DCM, 86%.

Two different approaches were followed for the introduction of the pyrene units in the PNA strands. The probe containing only 1 pyrene unit (**PNA1**, Figure 1c) was synthesized by on-resin modification of 5-azidomethyluracil precursor, as described previously [35], whereas a pyrene-containing modified monomer **1** (Scheme 1), more suitable for automated synthesis, was designed for the realization of all the other oligomers (**PNA2**–**6,** Figure 1c).

For the synthesis of the modified monomer bearing the pyrene moiety, we started from the 5-azidomethyluracil building block **2** previously synthesized by our lab [35]. The azide function was first reduced under Staudinger conditions to the corresponding amine **3**, on which 1-pyreneacetic acid was linked using HBTU/DIPEA as condensing agent. The ester **4** was then hydrolyzed to the acid **5**, and linked to the Fmoc-protected PNA backbone using EDC/DhBtOH as activating mixture; the PNA monomer **1**, was then obtained by ester hydrolysis of **6** under acidic conditions.

The PNAs sequence was designed to be complementary to the W1282X mutated form of CFTR gene, and all PNAs were synthesized using standard Fmoc-based manual solid phase protocol. The crude products were purified by RP-HPLC and characterized by HPLC–UV–MS (Supporting Information File 1, Figures S6–S11).

### Thermal stability of PNA:DNA complexes

The introduction of a modification in a PNA stand can lead to different effects, electronic or steric, which affect both self-aggregation of the PNA and their interactions with complementary DNA strand. Substitution at the C-5 position of the uracil ring allows positioning of the substituent in the direction of the major groove of the double helix, thus reducing the destabilization induced by steric factors; moreover the large aromatic portion introduced with the pyrene ring can interact with the flanking bases of the strand through π–π stacking interactions, thus stabilizing the complex formed.

For the evaluation of the sum of all this effects we measured the melting temperatures of the complexes formed between the PNAs and the full matched **DNA1** or the single mismatched **DNA2** (corresponding to the wild type CFTR gene), using both UV (Table 1, and Supporting Information File 1, Figures S12–S14) and fluorescence (Supporting Information File 1, Figure S15). The stability of these complexes was indeed found to be strongly dependent on the presence, the positioning and the number of pyrene units within the PNA strand.

**Table 1:** UV melting temperature of PNA:DNA complexes. All measurements were done in PBS at pH 7 with 1 μM strand concentration except for unmodified PNA measurements (5 μM strand concentration).

PNA	*T*_m_ PNA:**DNA1** (°C)	*T*_m_ PNA:**DNA2** (°C)	Δ*T*_m_ (°C)

**PNA1**	26	20	6
**PNA2**	39	19	20
**PNA3**	24	< 18	n.d.
**PNA4**	33^a^	22	11
**PNA5**	28	22	6
**PNA6**	n.d.^b^	n.d.^b^	n.d.
**PNA7**	34	24	10

^a^Broad transition observed. ^b^Continuous drift, no net transition observed.

The presence of a single pyrene unit (**PNA1**) destabilizes the PNA:DNA complex. The introduction of a second pyrene unit adjacent to the first one (**PNA3**) results in a further destabilization, whereas distal positioning of pyrene units (**PNA2**, **4**, **5**) leads to stabilization if compared to **PNA1**, but to an extent depending on the position of the second pyrene unit. For **PNA2** the additional interactions lead to the highest stability and very high selectivity, with Δ*T*_m_ strongly increased compared to the unmodified **PNA7** (20 °C vs 10 °C). The presence of a second pyrene unit at N-terminal position is more stabilizing than that at C-term (compare **PNA5** and **PNA2, 4**). **PNA4** is characterized by a broad melting curve, whereas for **PNA6** a continuous drift was observed already for the PNA alone, and in the presence of DNA no clear-cut transition was detected, suggesting a pyrene-mediated strong aggregation of the probe itself.

**Figure 2:**
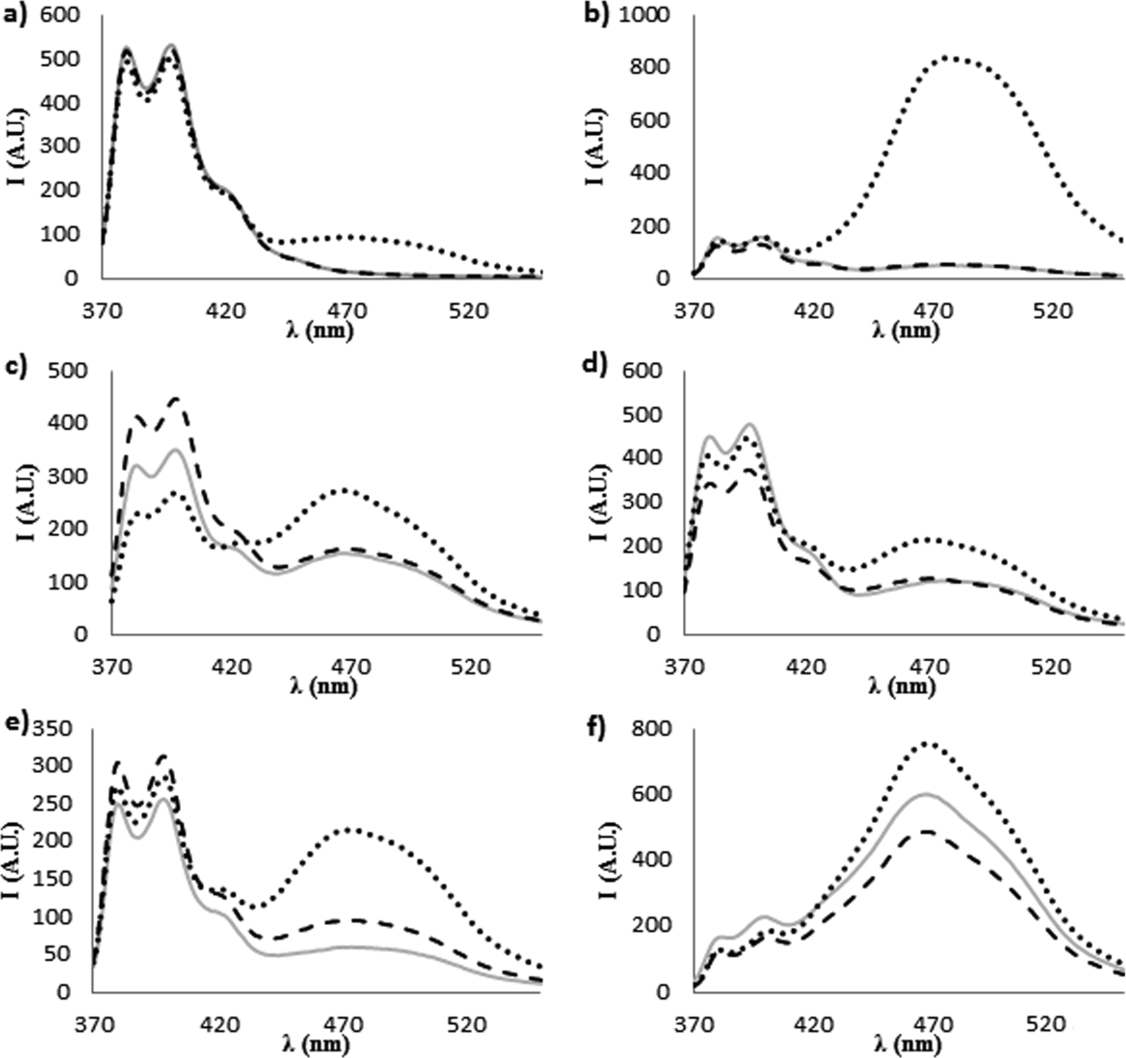
Fluorescence spectra at 347 nm excitation, recorded at 20 °C of: (a) **PNA1**, (b) **PNA2**, (c) **PNA3**, (d) **PNA4**, (e) **PNA5**, (f) **PNA6**. All measurements were done in PBS buffer, pH 7; concentration of each strand was 1 μM. Full lines are for ssPNA solutions, dotted lines are for PNA:**DNA1** solutions and broken lines are for PNA:**DNA2** solutions.

As described below, all the probes showed excimer emission in the 460–480 nm range upon hybridization (Figure 2). The temperature dependence of the excimer band in the presence of DNA was found to be in accordance with the UV melting measurements (Supporting Information File 1, Figure S15).

### Fluorescence studies

Beside the modification of thermal stability and selectivity induced by the incorporation of pyrene moieties described above, we evaluated the fluorescence properties of these PNA in the absence and in the presence of DNA. The evaluation of the pyrene quantum yields showed that these probes are much less fluorescent than the 1-pyreneacetic acid precursor in water (23 times lower quantum yield for **PNA2**, see Supporting Information File 1, Figure S16), probably due to the quenching effect of nucleobase units; however, the most important data are related to changes in the fluorescence spectrum upon hybridization with DNA, since this property is strongly related to the environment around the fluorophores [38] and can reveal interactions between pyrene units in the PNA:DNA complexes.

In Figure 2 the fluorescence emission spectra of the PNA probes in the absence or in the presence of complementary **DNA1** or mismatched **DNA2** are reported.

**Figure 3:**
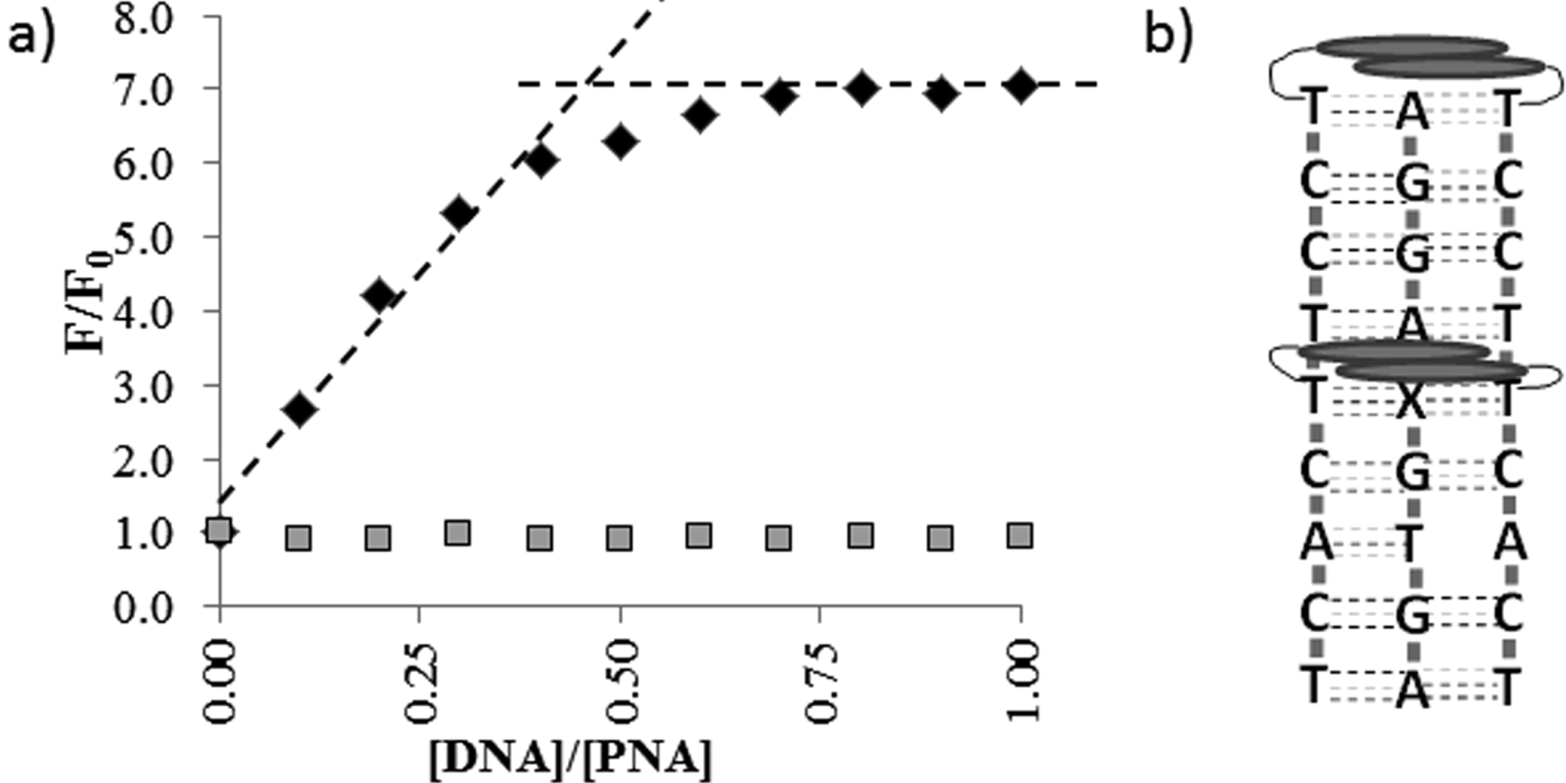
(a) Increase in fluorescence intensity of the excimer band for **PNA2** upon addition of complementary **DNA1** (black diamonds) or mismatched **DNA2** (grey squares) at 25 °C); (b) model of interaction showing both base recognition through hydrogen bonding and stacking interactions. X = A for full match, G for mismatch.

**Figure 4:**
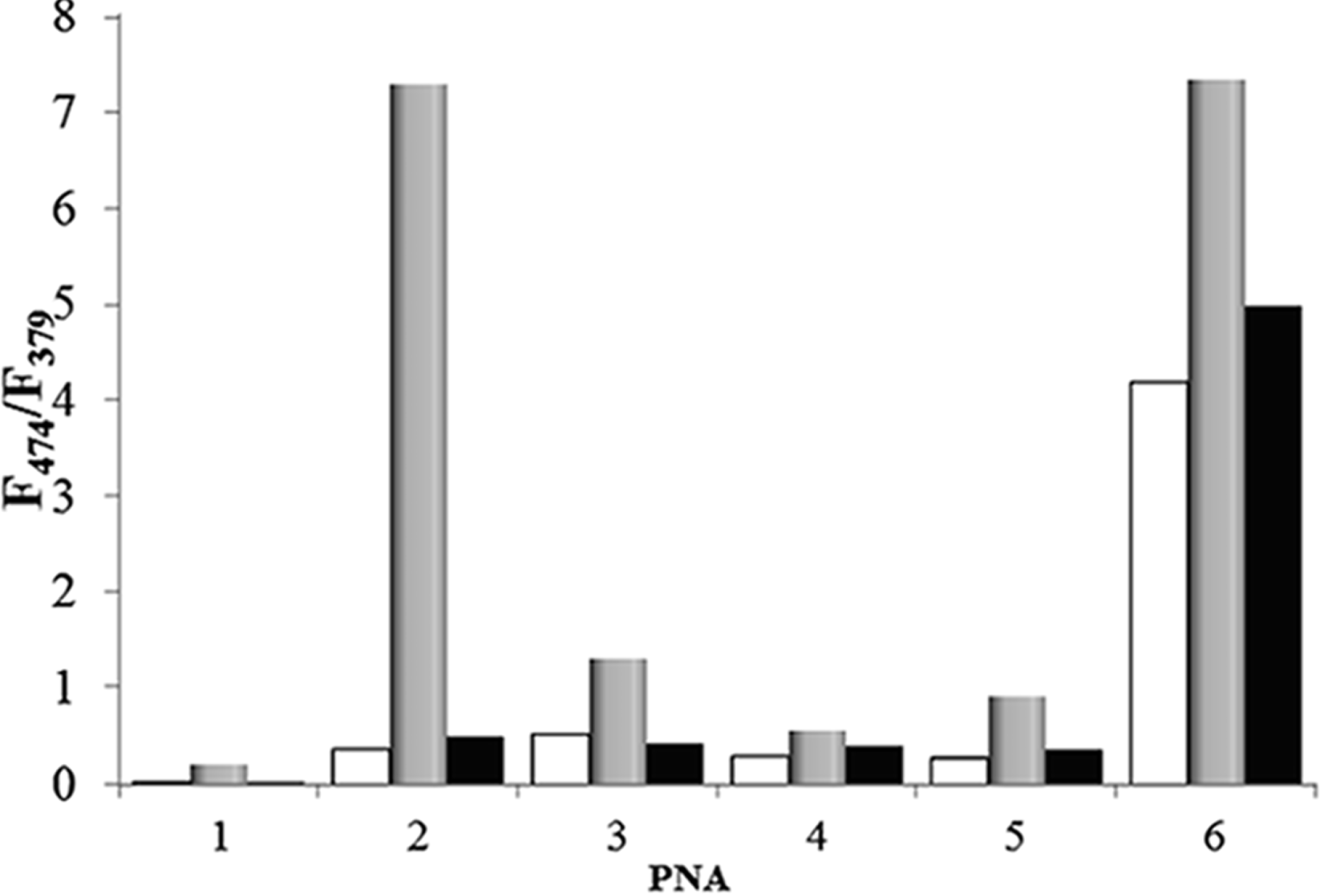
Ratio of the intensities of the pyrene excimer (F_474_) and monomer emission (F_379_) for the PNA probes in the absence of DNA (white bars), in the presence of **DNA1** (full match, grey bars), and **DNA2** (mismatch, black bars). Experiments were done at 20 °C in PBS at 1 μM PNA concentration (0.5 μM DNA concentration).

For **PNA1**, having only one pyrene unit, a typical pyrene excimer band was observed in the presence of **DNA1** (Figure 2a); this band cannot evidently derive from an intramolecular excimer and thus it must be due to a DNA-templated association of two PNA units. Furthermore, the same band was not observed in the presence of **DNA2**, thus indicating that the excimer formation is sequence-specific. **PNA2**, which has two distant pyrene units, showed a weak excimer emission, due to weak self-association (this band tend to disappear with dilution, see Supporting Information File 1, Figure S16), which underwent a dramatic enhancement when **PNA2** was bound to **DNA1**, whereas it remained unchanged by addition of **DNA2** (Figure 2b and Figure 3). This resulted in a very high increase in the excimer to monomer emission ratio (Figure 4), which can be exploited for analytical purposes in the case of the biologically relevant **DNA1** (mutated form) and **DNA2** (wilt type). The fluorescent responses for the other two mismatched DNA (**DNA3**: 5’-AGTG**C**AGGA-3’ and **DNA4**, 5’-AGTG**T**AGGA-3’), were also measured (Supporting Information File 1, Figure S18) and were shown to give rise to results comparable to that of **DNA2**. Accordingly, no melting transitions were observed for **PNA2** with **DNA3** and **DNA4** above 18 °C (data not shown). Thus the intensity of the excimer band was found to follow the expected sequence selectivity of the hydrogen-bonding scheme.

**PNA3** has two proximal pyrene units on adjacent bases, and therefore the free probe already shows excimer emission; this band was enhanced in the presence of the templating **DNA1**, whereas in the presence of the mutated **DNA2** the excimer band remained as in single strand and only a slight enhancement in the monomer emission was observed; thus, the excimer to monomer emission ratio (Figure 4) was slightly reduced. **PNA4** and **PNA5** showed an increase in the excimer fluorescence intensity signal upon hybridization with the templating DNA, though lower than for **PNA2**. **PNA6** has already a strong excimer emission as single-strand, but this band was slightly enhanced upon interaction with the full-match **DNA1**, whereas it was slightly reduced in the presence of **DNA2**.

For all PNAs, very similar results were obtained in fluorescence response induced by **DNA3** and **DNA4**, except that for **PNA6** the difference observed with **DNA4** was less pronounced than with other mismatches (Supporting Information File 1, Figure S18).

The DNA-induced formation or enhancement of these excimer bands can be explained if a PNA_2_DNA triplex is formed, favoured by the prevalence of pyrimidines in the PNA [22–23]. The PNA_2_DNA triplex, in this sequence, is destabilized by the presence of a pyrimidine base (T) in the 5’-end of the DNA; thus this sequence represents a good model for evaluating the stabilization/destabilization effects due to the presence of pyrene units. The nature of these PNA:DNA complexes was confirmed by titration experiments; for **PNA7** CD titrations revealed a 2:1 stoichiometry (Supporting Information File 1, Figure S19); the same stoichiometry was found for **PNA2** by following the increase in the excimer emission as a function of DNA concentration (Figure 3a).

The hysteresis observed between melting and annealing curves (Supporting Information File 1, Table S1) is also indicative of the formation of triplex structures between the PNAs and the DNA.

### Evaluation of pyrene-modified **PNA2** as fluorescent probe

In Figure 4 the ratios between the excimer and monomer emission of each probe alone and in the presence of 0.5 equivalents of the full match **DNA1** and mismatched **DNA2** are reported. Under these conditions, **PNA1** showed an increased, though weak, excimer emission, whereas **PNA2** showed a dramatic increase in the presence of **DNA1** and very low one in the presence of **DNA2**. Thus, **PNA2** showed best performances in terms of excimer signal intensity ΔF_FM_ (difference between the fluorescence in the presence of full match **DNA1** and that of single strand PNA) and of selectivity compared to the wild-type mismatch (MM) reaching ΔF_FM_/ΔF_MM_ = 180.

Using a 1 μM **PNA2** solution we calculated the limit of detection (LOD) of the fluorescence detection of **DNA1** using this probe. A linear regression was obtained in the low nanomolar range, and a LOD of 18.7 nM for **DNA1** was calculated (see Supporting Information File 1).

### Effect of pyrene units on stability and sensing

According to the occurrence of excimer bands in the fluorescence spectra of the PNA probes (Figure 2 and Figure 4) the presence of pyrene favours self-association of two PNA strands; strong interactions should be observed for **PNA2**–**5** and even stronger for **PNA6**, i.e., with the increase of the number of the pyrene units, as indeed experimentally observed. The following model can be used to rationalise the observed data for DNA interaction. The stacking interactions (Figure 1a) occurring between pyrene units of different PNA strands (schematically depicted in Figure 3b for **PNA2**) affect triplex formation, which is also biased by both steric and conformational effects; the base pairings of the adenines in the target DNA with the modified uracil units allow the two pyrene residues to be kept close enough to interact (generating an excimer band), but this process can result in destabilization of the overall structure (see *T*_m_ of **PNA1** in Table 1). However, for **PNA2**, the combined effect of two pyrene pairs properly positioned allows to increase both stability and selectivity of PNA compared to unmodified one. The N-terminal pyrene unit, in addition to the central one (which has the same position as in **PNA1**), stabilizes the triplex structure through the occurrence of combined stacking interactions (Figure 3b). Thus, the presence of a single mismatch facing the central modified monomer results in destabilization not only of the excimer corresponding to this nucleobase, but of the entire triplex, leading to high mismatch recognition. This induces the very high selectivity in the switch-on of the excimer fluorescence emission (Figures 2, 3 and 4). All the other tested dispositions are not so effective in terms of stabilization, fluorescence response and selectivity; for **PNA3** this is attributable to steric hindrance between the adjacent pyrene units; **PNA4** and **PNA5** containing one pyrene unit in the C-term, at the end of a segment in which the triplex structure is destabilized by the presence on the PNA of one adenine unit, show less selectivity; furthermore a gradual transition was observed for **PNA4**, suggesting weak cooperativity in the stacking interaction. The presence of three pyrene residues (**PNA6**) instead, induce a strong self-aggregation of the PNA alone; this assembling process is favoured by the presence of **DNA1** and to a lesser extent **DNA2** (Figure 2 and Figure 4a); however, the strong excimer signal of the PNA alone prevents its use as an efficient probe for DNA.

## Conclusion

In conclusion, we have demonstrated that introduction of two pyrene units protruding into the major groove and properly positioned along the PNA strand (as in **PNA2**) can stabilize PNA_2_DNA triplex structures by additional stacking interactions which combine with Watson–Crick and Hoogsteen base pairing; these interactions are clearly detectable by the formation of the excimer band of pyrene in the fluorescence spectra. Thus this work makes a significant step toward the objective of stabilizing triplexes even in the presence of pyrimidines on the target sequence, while still maintaining and even increasing sequence selectivity. Moreover, for diagnostics, it is important that a very high and sequence-selective excimer to monomer ratio can be obtained, as with **PNA2**, upon hybridization, a property which is very important also in the case of more elaborated methods such as gated detection. These characteristics make **PNA2** a very good fluorescent probe, with very high single-base selectivity in both thermal stability and excimer formation upon binding to target DNA. Thus, the present results can be very useful in the design of new probes for single point mutations and single nucleotide polymorphisms (SNPs), highly relevant in the genomic as well as in the clinical fields.

## Experimental

### General information

Reagents were purchased from Sigma-Aldrich, Fluka, Merck, Carlo Erba, TCI Europe, Link, ASM and used without further purification. All reactions were carried out under a nitrogen atmosphere with dry solvents under anhydrous conditions, unless otherwise noted. Anhydrous solvents were obtained by distillation or anhydrification with molecular sieves. Reactions were monitored by TLC carried out on 0.25 mm E. Merck silica-gel plates (60F-254) by using UV light as visualizing agent and ninhydrin solution and heat as developing agents. E. Merck silica gel (60, particle size 0.040–0.063 mm) was used for flash-column chromatography. NMR spectra were recorded on Bruker Avance 400 or 300 instruments and calibrated by using residual undeuterated solvent as an internal reference. The following abbreviations were used to explain the multiplicities: s = singlet, d = doublet, t = triplet, q = quartet, m = multiplet and br = broad. IR spectra were measured using a FTIR Thermo Nicolet 5700, in transmission mode using KBr or NaCl. HPLC–UV–MS were recorded by using a Waters Alliance 2695 HPLC with Micromass Quattro microAPI spectrometer, a Waters 996 PDA and equipped with a Phenomenex Jupiter column (250 × 4.6 mm, 5 μm, C18, 300 Å) (method A, 5 minutes in H_2_O 0.2% formic acid (FA), then linear gradient to 50% MeCN 0.2% FA in 30 minutes at a flow rate of 1 mL/min). PNA oligomers were purified with RP-HPLC using a XTerra Prep RP_18_ column (7.8 × 300 mm, 10 μm) (method B, linear gradient from H_2_O 0.1% TFA to 50% MeCN 0.1% TFA in 30 minutes at a flow rate of 4.0 mL/min). HRMS were recorded using a Thermo LTQ-Orbitrap XL.

**Synthesis and characterization of compounds 1, 3–6** are reported in Supporting Information File 1.

**Synthesis and characterization of PNAs.** The synthesis of **PNA1** was already described in a previous work [35]. The syntheses of all the other PNAs, bearing multiple pyrene units (**PNA2**, **PNA3**, **PNA4**, **PNA5** and **PNA6**), were performed with standard Fmoc-based manual synthesis protocol using **1** in addition to standard monomers, on a Rink amide resin loaded with Fmoc-Gly-OH as first monomer (0.2 mmol/g). The unmodified **PNA7** was synthesized using a standard Boc-based manual protocol using commercial monomers on a MBHA resin loaded with Fmoc-Gly-OH as first monomer (0.2 mmol/g). PNA purifications were performed by RP-HPLC with UV detection at 260 nm (gradient B). The purity and identity of the purified PNAs were determined by HPLC–UV–MS (gradient A). **PNA2**: 9%; *t*_r_: 24.7 min; ESI-MS (*m*/*z*): calcd for [M]: 2932.1426; found: 1466.9 [MH_2_]^2+^, 978.2 [MH_3_]^3+^, 733.9 [MH_4_]^4+^, 587.2 [MH_5_]^5+^; **PNA3**: 10%; *t*_r_: 24.6 min; ESI-MS: (*m*/*z*): calcd for [M]: 2932.1426; found: 1466.9 [MH_2_]^2+^, 978.2 [MH_3_]^3+^, 733.9 [MH_4_]^4+^, 587.3 [MH_5_]^5+^; **PNA4**: 11%; *t*_R_: 23.7 min; ESI-MS (*m*/*z)*: calcd for [M]: 2932.1426; found: 1466.8 [MH_2_]^2+^, 978.2 [MH_3_]^3+^, 733.9 [MH_4_]^4+^, 587.3 [MH_5_]^5+^; **PNA5**: 15%; *t*_R_: 24.0 min; ESI-MS (*m*/*z*): calcd for [M]: 2932.1426; found: 1467.1 [MH_2_]^2+^, 978.2 [MH_3_]^3+^, 733.8 [MH_4_]^4+^, 587.3 [MH_5_]^5+^; **PNA6**: 11%; *t*_R_: 27.4 min; ESI-MS (*m*/*z*): calcd for [M]: 3189.2266; found: 1064.1 [MH_3_]^3+^, 798.2 [MH_4_]^4+^, 638.7 [MH_5_]^5+^; **PNA7**: 25%; *t*_R_: 18.5 min; ESI-MS (*m*/*z*): calcd for [M]: 2419.4159; found: 1210.4 [MH_2_]^2+^, 807.3 [MH_3_]^3+^, 605.7 [MH_4_]^4+^, 484.8 [MH_5_]^5+^. Yields reported in % for each PNA are those of purified products, calculated by UV–vis analysis.

**UV measurements**. Stock solutions of PNA and DNA synthetic oligonucleotides (Thermo-Fisher Scientific, HPLC-grade) were prepared in double-distilled water, and the PNA concentration was calculated by UV absorbance with the following extinction coefficients (ε_260_ [M^–1^cm^–1^]) for the nucleobases: T 8600, T* 14938 (pyrene-modified monomer, see Supporting Information File 1, Figure S12 for the calculation of this value), C 6600, A 13700, and G 11700. For DNA the data provided by the producer were used. From these, solutions containing single stranded PNA and DNA or PNA:DNA duplexes were prepared. Measurement conditions: [PNA] = [DNA] = 1 μM in PBS (100 mM NaCl, 10 mM NaH_2_PO_4_·H_2_O, 0.1 mM EDTA, pH 7.0, 1.25% DMF). All the samples were first incubated at 90 °C for 5 min, then slowly cooled to room temperature. Thermal denaturation profiles (A_260_ versus *T*) of the hybrids were measured with a UV–vis Lambda Bio 20 spectrophotometer equipped with a Peltier temperature programmer PTP6 interfaced to a personal computer. For the temperature range 18–50 °C, A_260_ values were recorded at 0.1 °C increments, with a temperature ramp of 1 °C/min. Both melting and annealing curves were recorded for each solution. The melting temperature (*T*_m_) was determined from the maximum of the first derivative of the melting curves.

**Fluorescence measurements.** Fluorescence spectra were recorded on a Perkin Elmer LS55 luminescence spectrometer equipped with a LAUDA ECOline RE104 temperature control system, exciting at 347 nm (slit: 5.0 nm), scanning from 370 nm to 550 nm, a scan speed of 200 nm/min was used and 3 accumulation for each spectrum. Samples were prepared as reported for UV measurements. From the stock solutions, described above, solutions of PNA alone (1 μM in PBS) and of PNA/DNA 2:1 (1 μM PNA and 0.5 μM DNA in PBS) were prepared. All the samples were first incubated at 90 °C for 5 min, and then slowly cooled to the temperature of analysis. Fluorescence emission spectra were recorded with an excitation wavelength of 347 nm (slit excitation: 5.0 nm), scanning from 370 nm to 550 nm (slit emission: 10.0 nm), a scan speed of 200 nm/min was used with 3 spectra accumulation for each solution. All measurements were compensated for lamp fluctuations by normalization using as reference a 20 nM 1-pyreneacetic acid solution in PBS. Equilibration of the solution and complete formation of the complexes were checked by repeating the analysis after 10 minutes, to ensure that no significant variation of the fluorescence profiles was present. Variable temperature fluorescence measurements are reported in Supporting Information File 1.

**Fluorescence titration of PNA2 and PNA3.** From the stock solution described above single stranded PNA solutions (1 μM in PBS) and single stranded DNA solutions (10 μM in PBS) were prepared. PNA solutions were first incubated at the experimental temperature, then spectra were recorded after addition of portions of DNA (10% of the PNA amount each), allowing an equilibration time of 8 min. Fluorescence emission spectra were recorded with an excitation wavelength of 347 nm (slit: 5.0 nm), scanning from 370 nm to 550 nm, with scan speed of 200 nm/min, and 3 spectra accumulation for each solution. All measurements were corrected for dilution, and compensated for lamp fluctuations by normalization using as reference a 20 nM 1-pyreneacetic acid solution in PBS. Fluorescence titration of **PNA3** at 10 °C is reported in Supporting Information File 1, Figure S17.

## Supporting Information

File 1 Synthesis, characterization, and spectral data of compounds **1, 3–6**, HPLC–MS analyses of **PNA1–7**, additional UV, fluorescence and CD data.
