# Supplementary material for: Pyrene-modified PNAs: Stacking interactions and selective excimer emission in PNA2DNA triplexes
Source: Beilstein J Org Chem. 2014 Jul 2;10:1495–503. doi: 10.3762/bjoc.10.154 (PMC4142857; doi:10.3762/bjoc.10.154)
Supplement: File 1 — Synthesis, characterization, and spectral data of compounds 1, 3–6, HPLC–MS analyses of PNA1–7, additional UV, fluorescence and CD data. [file Beilstein_J_Org_Chem-10-1495-s001.pdf]

## Supporting Information

## for

# Pyrene-modified PNAs: Stacking interactions and selective excimer emission in $PNA_2DNA$ triplexes


Alex Manicardi*[1], Lucia Guidi,[1] Alice Ghidini[1,2] and Roberto Corradini*[1]

Address: [1]Department of Chemistry, University of Parma, Parco Area delle Scienze 17/A, 43124, Parma, Italy. Fax: +39 0521 905472; Tel: +39 0521 905410, and [2]Present Address: Department of Biosciences and Nutrition, Karolinska Institutet, Novum, Hälsovägen 7, 14183, Huddinge, Sweden.

Email: Roberto Corradini* - roberto.corradini@unipr.it; Alex Manicardi* - alex.manicardi@unipr.it

*Corresponding author


## Synthesis, characterization, and spectral data of compounds 1, 3–6, HPLC–MS analyses of PNA1–7, additional UV, fluorescence and CD data

*Contents*


**1. Synthesis of modified monomer 1** **s3–6**

**2. Spectra of synthesised compounds 3–6 and 1** **s7–11**

Figure S1: $^{1}$H NMR and $^{13}$C NMR of compound **3** s7

Figure S2: $^{1}$H NMR, $^{13}$C NMR and HRMS of compound **4** s8

Figure S3: $^{1}$H NMR, $^{13}$C NMR and HRMS of compound **5** s9

Figure S4: $^{1}$H NMR, $^{13}$C NMR and HRMS of compound **6** s10

Figure S5: $^{1}$H NMR, $^{13}$C NMR and HRMS of compound **1** s11

**3. Characterization of PNA 2–7** **s12–17**

Figure S6: HPLC–MS analysis of **PNA2**. s12

Figure S7: HPLC–MS analysis of **PNA3**. s13

Figure S8: HPLC–MS analysis of **PNA4**. s14

Figure S9: HPLC–MS analysis of **PNA5**. s15

Figure S10: HPLC–MS analysis of **PNA6**. s16

Figure S11: HPLC–MS analysis of **PNA7**. s17

**4. UV measurements** **s18–20**

**4.1 Calculation of the extinction coefficient for 5** **s18**

Figure S12: Linear regression for the evaluation of the extinction coefficient of 5 s18

**4.2 Variable temperature UV measurements** **s19–20**

Figure S13: Relative absorbance at 260 nm as a function of temperature for PNA:DNA1 complexes s19

Figure S14: Relative absorbance at 260 nm as a function of temperature for PNA:DNA2 complexes s19

Table S1: UV melting and annealing temperatures of PNA:DNA complexes s20

**5. Fluorescence measurements** **s21–23**

**5.1 Fluorescence analysis at variable temperature** **s21**

Figure S15: Fluorescence intensities as a function of temperature s21

**5.2 Relative Fluorescence of PNA2 vs 1-pyreneacetic acid** **s22**

Figure S16: Emission spectra of PNA 2 and of 1-pyreneacetic acid s22

**5.3 Fluorescence titration of PNA3** **s23**

Figure S17: Fluorescence titration PNA3 s23

**5.4 Determination of the limit of detection (LOD) for DNA1.** **s23**

**5.5 Base selectivity** **s24**

**6. Circular dichroism titration of PNA7** **s25**

Figure S19: CD titration of DNA FM with PNA 7 s25

## 1. Synthesis of modified monomer 1

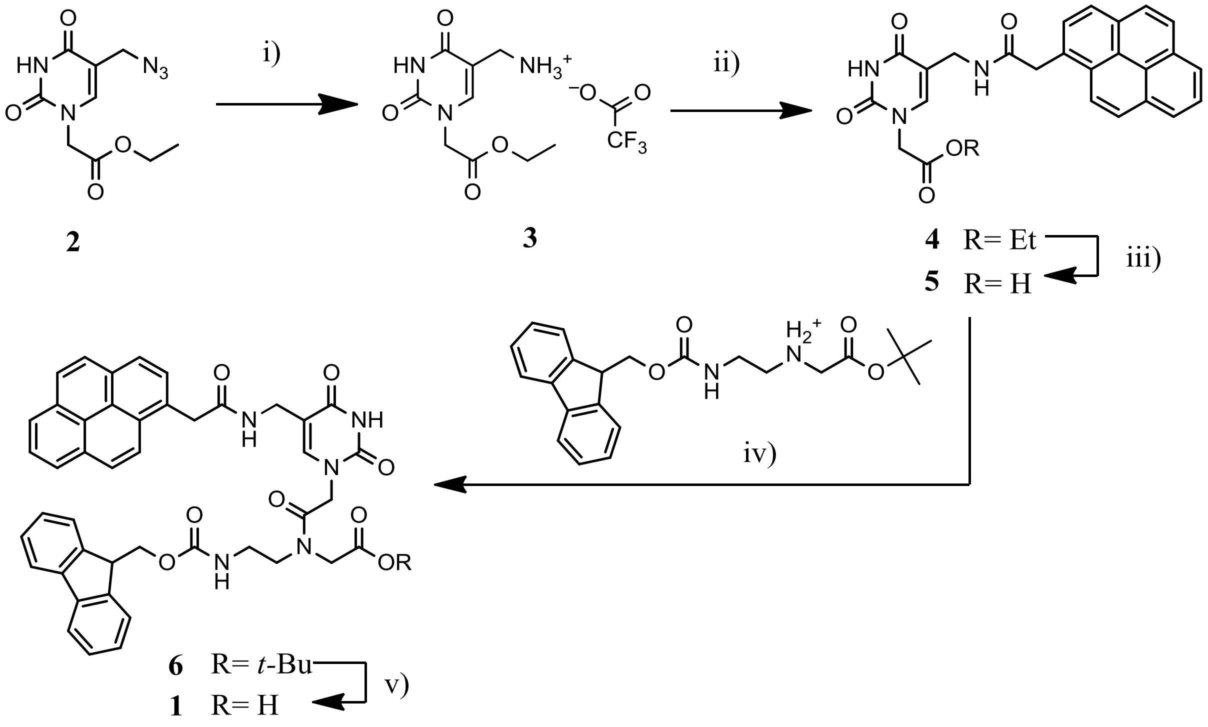

**Ethyl 2-(5-aminomethyluracil-1-yl)acetate trifluoroacetate salt (3).** In a round bottom flask ethyl 2-(5-azidomethyluracil-1-yl)acetate [1] (1.10 g, 4.33 mmol) was solubilized in 20 mL THF, then water (0.156 mL, 8.67 mmol) was added and the reaction mixture was cooled to 0 °C with an ice bath before the addition of triphenylphosphine (1.70 g, 6.50 mmol). The reaction was left to warm to rt and to react overnight. The solvent was then evaporated under reduced pressure, the crude product was taken up with a minimum amount of TFA then precipitated with $Et_2O$, collected by centrifugation and washed with DCM (3 times) to yield **3** as white solid (0.70 g, 71%). TLC (AcOEt/MeOH/$NH_{3\,(aq)}$ 5:4:1) Rf: 0.32; MP (°C): 113.8-115.2; $^1$H NMR (DMSO-d$^6$, 300 MHz) δ (ppm): 11.84 (1H, s), 7.99 (3H, br s), 7.88 (1H, s), 4.56 (2H, s), 4.16 (2H, q, J= 7.1 Hz), 3.65 (2H, d, J= 5.0 Hz), 1.21 (3H, t, J= 7.1 Hz); $^{13}$C NMR (DMSO-d$^6$, 75 MHz) δ (ppm): 167.7, 163.2, 158.2 (q, J= 34 Hz), 150.4, 146.1, 116.3 (q, J= 296 Hz), 105.7, 61.1, 48.8, 35.1, 13.8; MS (ESI, MeOH): *m/z* calcd for $C_9H_{13}N_3O_4$ [M]: 227.0906, found: 228.2 $[M+H]^+$, 455.4 $[2M+H]^+$; Elemental

composition: calcd %C 38.72, %H 4.14, %N 12.31, found %C38.85, %H 4.40, %N 12.28; FT-IR (KBr) n ($cm^{-1}$): 3566 (m), 3446 (m), 3200 (m), 2998 (m), 2829 (m), 1728 (s), 1713 (s), 1667 (s).

**Ethyl 2-(5-(2-(pyren-1-yl)acetamido)methyluracil-1-yl)acetate (4).** In a round bottom flask 1-pyreneacetic acid (305 mg, 1.17 mmol) and HBTU (434 mg, 1.15 mmol) were dissolved in 5 mL DMF, the solution was then cooled to 0 °C with an ice bath and DIPEA was added (485 µL, 2.94 mmol). The mixture was left to react for 30 minutes at 0 °C, then to warm to rt. After 30 minutes **3** (200 mg, 0.59 mmol) was added and the mixture was left to react overnight. **4** was then collected as a beige solid over a Büchner funnel after precipitation from the reaction mixture with 20 mL AcOEt and 20 mL $H_2O$. A second aliquot of product was collected by precipitation with 20 mL hexane from the organic layer washed with saturated $KHSO_4$ (2 × 50 mL), saturated $NaHCO_3$ (2 × 50 mL) and brine (50 mL). Yield: 183 mg, 66%. TLC (AcOEt/MeOH/$NH_3$(aq) 5:4:1) Rf: 0.33; MP (°C): decompose without melting at 226°C; $^1$H NMR (DMSO-$d^6$, 400 MHz) δ (ppm): 11.57 (1H, s), 8.53 (1H, t, J= 5.5 Hz), 8.41 (1H, d, J= 9.3 Hz), 8.29 (2H, d, J= 7.5 Hz), 8.24 (1H, d, J= 7.8 Hz), 8.20 (1H, d, J= 9.3 Hz), 8.15 (2H, s), 8.07 (1H, d, J= 7.6 Hz), 8.02 (1H, d, J= 7.9 Hz), 7.53 (1H, s), 4.46 (2H, s), 4.24 (2H, s), 4.10 (2H, q, J= 7.1 Hz), 3.92 (2H, d, J= 5.4 Hz), 1.17 (3H, t, J= 7.1 Hz); $^{13}$C NMR (DMSO-$d^6$, 100 MHz) δ (ppm): 170.7, 168.4, 163.9, 151.2, 143.5, 131.3, 131.4, 130.8, 130.2, 129.5, 129.1, 127.8, 127.7, 127.3, 126.6, 125.6, 125.4, 125.2, 124.6, 124.4, 110.7, 61.6, 49.2, 40.4, 35.7, 14.4; MS (ESI, MeOH): *m/z* calcd for $C_{27}H_{23}N_3O_5$ [M]: 469.16377, found: 470.4 $[M+H]^+$, 492.3 $[M+Na]^+$, 508.3 $[M+K]^+$, 468.4 $[M-H]^-$, 504.4 $[M+Cl]^-$; HRMS (MeOH): *m/z* calcd for $[C_{27}H_{24}N_3O_5]^+$: 470.17105, found: 470.17075; FT-IR (KBr) n ($cm^{-1}$): 3041 (m), 1670 (s), 1684 (s), 1472 (w).

**2-(5-(2-(Pyren-1-yl)acetamido)methyluracil-1-yl)acetic acid (5).** In a round bottom flask **4** (248 mg, 0.53 mmol) was dissolved in 10 mL MeOH, 5 mL 1 M NaOH were added and the mixture and left to react overnight. The organic solvent was then evaporated under vacuum, the pH was lowered

to 3 with 37% HCl and **5** was collected over a Büchner funnel as a red solid. Yield: 212 mg, 91%. TLC (AcOEt) Rf: 0.00 (check for the absence of **4**); MP (°C): decompose without melting at 165°C; $^1$H NMR (DMSO-d$^6$, 400 MHz) δ (ppm): 13.15 (1H, br s), 11.52 (1H, s), 8.51 (1H, br t, J= 5.4 Hz), 8.40 (1H, d, J= 9.3 Hz), 8.29 (2H, d, J= 7.6 Hz), 8.24 (1H, d, J= 7.9 Hz), 8.20 (1H, d, J= 9.3 Hz), 8.15 (2H, s), 8.08 (1H, d, J= 7.6 Hz), 8.02 (1H, d, J= 7.9 Hz), 7.56 (1H, s), 4.39 (2H, s), 4.25 (2H, s), 3.92 (2H, d, J= 5.2 Hz); $^{13}$C NMR (DMSO-d$^6$, 100 MHz) δ (ppm): 170.7, 169.9, 164.0, 151.2, 143.7, 131.4, 131.3, 130.8, 130.2, 129.5, 129.1, 127.8, 127.7, 127.3, 126.6, 125.5, 125.4, 125.2, 124.6, 124.4, 110.5, 49.3, 49.2, 35.8; MS (ESI, MeOH): *m/z* calcd for $C_{25}H_{19}N_3O_5$ [M]: 441.13247, found: 442.2 $[M+H]^+$, 464.3 $[M+Na]^+$, 480.2 $[M+K]^+$, 905.6 $[2M+Na]^+$, 440.3 $[M-H]^-$, 881.6 $[2M-H]^-$, 903.6 $[2M+Na-2H]^-$; HRMS (MeOH) *m/z* calcd for $[C_{25}H_{18}N_3O_5]^-$: 440.12579, found: 440.12518; FT-IR (KBr) n (cm$^{-1}$): 3041 (m), 1700 (s), 1684 (s), 1472 (w).

***tert*-Butyl 2-(*N*-(2-Fmoc-aminoethyl)-2-(5-(2-(pyren-1-yl)acetamido)methyluracil-1-yl)acetamido)acetate (6).** Following a similar procedure described in [35], in a round bottom flask **5** (200 mg, 0.452 mmol) was solubilized in 2 mL dry DMF at 0 °C together with EDC·HCl (104 mg, 0.543 mmol), DhBtOH (74 mg, 0.453 mmol) and DIPEA (179 µL, 1.086 mmol) and left to react for 10 minutes before warming to rt. After 20 minutes *tert*-butyl 2-((2-Fmoc-aminoethyl)amino)acetate hydrochloride (104 mg, 0.543 mmol) was added and the reaction mixture was stirred for further 4 h. The reaction was then diluted with 200 mL AcOEt and washed with saturated $KHSO_4$ (2 × 200 mL), saturated $NaHCO_3$ (2 × 200 mL) and brine (200 mL). The organic fraction was dried over $Na_2SO_4$ and concentrated under vacuum. Flash chromatography (from AcOEt to AcOEt/MeOH 9:1) yielded **6** as pale brown solid. Yield: 252 mg, 68%. TLC (AcOEt/MeOH 7:3) Rf: 0.71; MP (°C): decompose without melting at 153°C; $^1$H NMR ($CDCl_3$, 400 MHz, major rotamer) δ (ppm): 9.67 (1H, s), 8.10÷8.03 (3H, m), 8.01÷7.95 (2H, m), 7.93÷7.85 (3H, m), 7.83÷7.75 (1H, m), 7.72 (2H, d, J= 7.3 Hz), 7.59 (2H, d, J= 7.3 Hz), 7.39÷7.32 (2H, m), 7.31÷7.21 (2H, m), 7.01 (1H, s), 6.67 (1H, t, J= 5.8 Hz), 6.04 (1H, t, J= 5.7 Hz), 4.42 (2H, d, J= 6.7

Hz), 4.25÷4.05 (5H, m), 3.90÷3.81 (4H, m), 3.44 (2H, br s), 3.26 (2H, br s), 1.44 (9H, s); $^{13}$C NMR ($CDCl_3$, 100 MHz, major rotamer) δ (ppm): 28.0, 36.3, 39.3, 41.5, 47.9, 48.9, 49.8, 66.8, 120.0, 123.1, 124.5, 125.0, 125.2, 126.0, 127.1, 127.2, 127.3, 127.7, 128.0, 128.5, 128.8, 129.5, 130.7, 131.1, 143.9, 150.5, 163.9, 167.7, 168.5, 171.2, 171.7; MS (ESI, MeOH): *m/z* calcd for $C_{48}H_{45}N_5O_8$ [M]: 819.32681, found: 842.5 $[M+Na]^+$, 858.5 $[M+K]^+$, 1663.0 $[2M+Na]^+$; HRMS (MeOH) *m/z* calcd for $[C_{48}H_{46}N_5O_8]^+$: 820.33409, found: 820.33386; FT-IR (KBr) n ($cm^{-1}$): 3414 (m), 3043 (w), 1676 (s), 1522 (m).

**2-(*N*-(2-Fmoc-aminoethyl)-2-(5-(2-(pyren-1-yl)acetamido)methyluracil-1-yl)acetamido)acetic acid (1).** Following a similar procedure described in [35], in a round bottom flask **6** (241 mg, 0.2941 mmol) was solubilized in 6 mL DCM at 0 °C, then TFA (4 mL) was added and the reaction mixture was left to react. After 5 minutes the reaction mixture was left to warm to rt and to react for further 2 h. The solvent was then co-evaporated with MeOH and $CHCl_3$ under reduced pressure. The resulting oil was dispersed in 20 mL $H_2O$ and filtered through a Büchner funnel to yield **1** as pale brown solid. Yield: 193 mg, 86%. TLC (AcOEt/MeOH 1:1) Rf: 0.49; MP (°C): decompose without melting at 166°C; $^1$H NMR (DMSO-d$^6$, 400 MHz, major rotamer) δ (ppm): 12.82 (1H, br s), 11.48 (1H, s), 8.52 (1H, br s), 8.41 (1H, s), 8.30÷8.25 (2H, m), 8.23 (2H, d, J= 9.0 Hz), 8.14 (1H, s), 8.09÷7.98 (2H, m), 7.88 (2H, d, J= 7.5 Hz), 7.67 (2H, d, J= 7.1 Hz), 7.40 (6H, t, J= 7.5 Hz), 4.66 (2H, br s), 4.30÷4.37 (3H, m), 4.29÷4.18 (4H, m), 3.92 (2H, br s), 3.44÷3.32 (2H, m), 3.29÷3.10 (2H, m); $^{13}$C NMR (DMSO-d$^6$, 100 MHz, major rotamer) δ (ppm): 47.2, 47.3, 48.2, 48.6, 49.5, 65.4, 66.0, 110.4, 120.6, 124.4, 124.6, 125.2, 125.4, 125.5, 125.6, 126.6, 127.3, 127.5, 127.7, 127.9, 128.1, 129.2, 129.5, 130.2, 130.8, 131.3, 131.4, 141.2, 144.3, 151.2, 151.8, 164.4, 167.9, 171.3; MS (ESI, MeOH): *m/z* calcd for $C_{44}H_{37}N_5O_8$ [M]: 763.26421, found: 786.5 $[M+Na]^+$, 802.3 $[M+K]^+$, 762.5 $[M-H]^-$; HRMS (MeOH) *m/z* calcd for $[C_{44}H_{36}N_5O_8]^-$: 762.25694, found: 762.25629; FT-IR (KBr) n ($cm^{-1}$): 3409 (m), 1684 (s), 1559 (m), 1472 (m).

# 2. Spectra of synthesised compounds 3–6 and 1

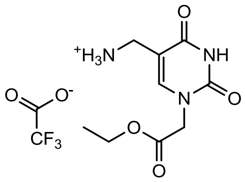


**3**

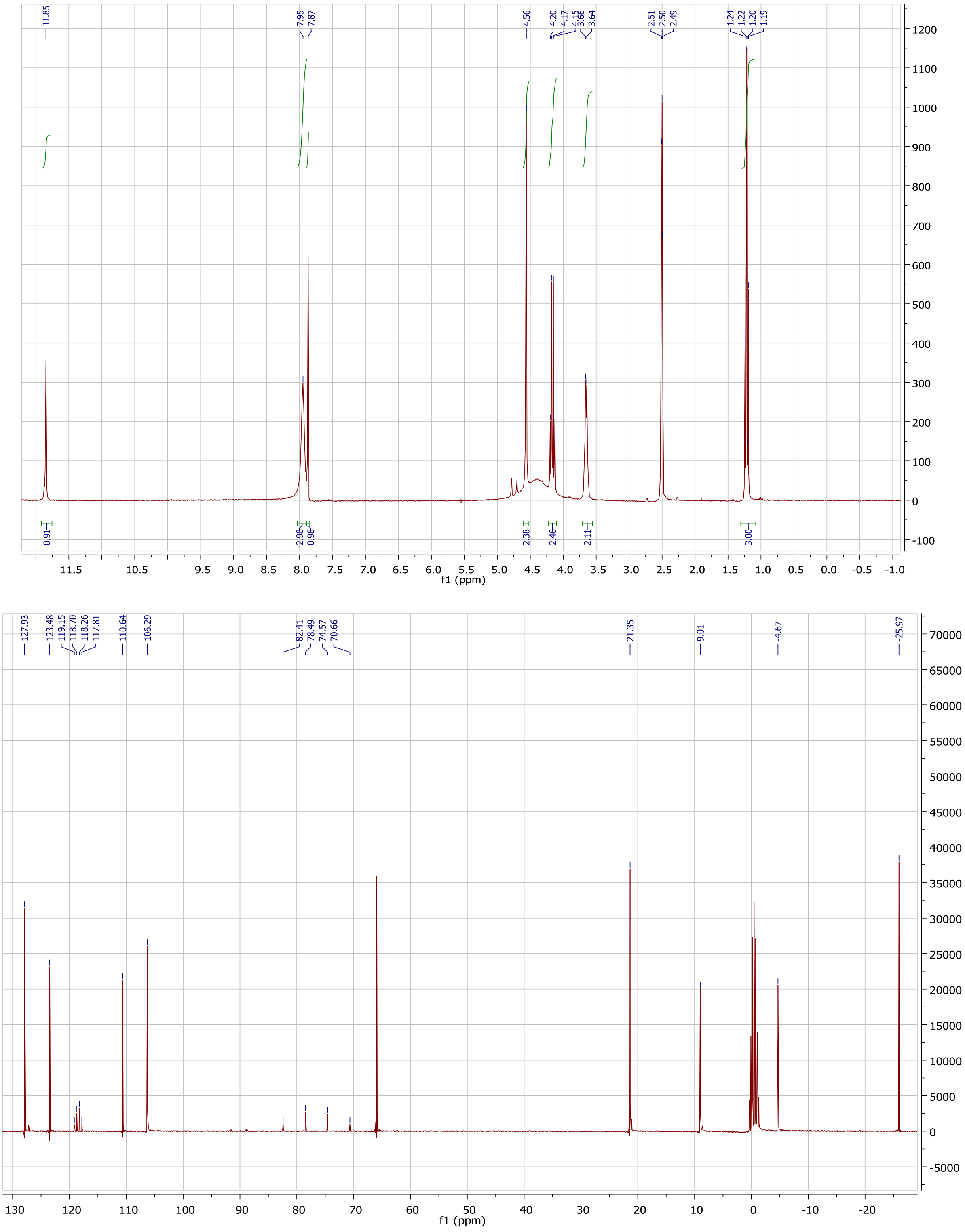


**Figure S1:** $^{1}$H NMR and $^{13}$C NMR of compound **3** (in DMSO-$d_6$).

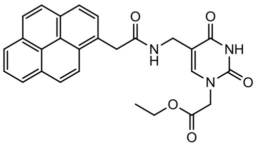


**4**

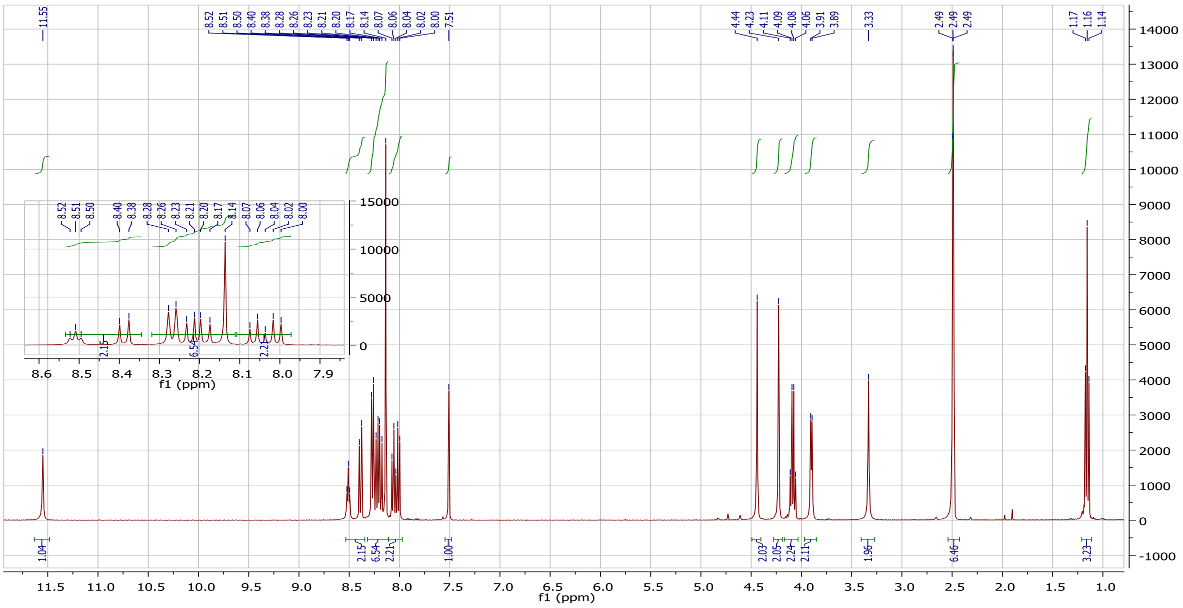


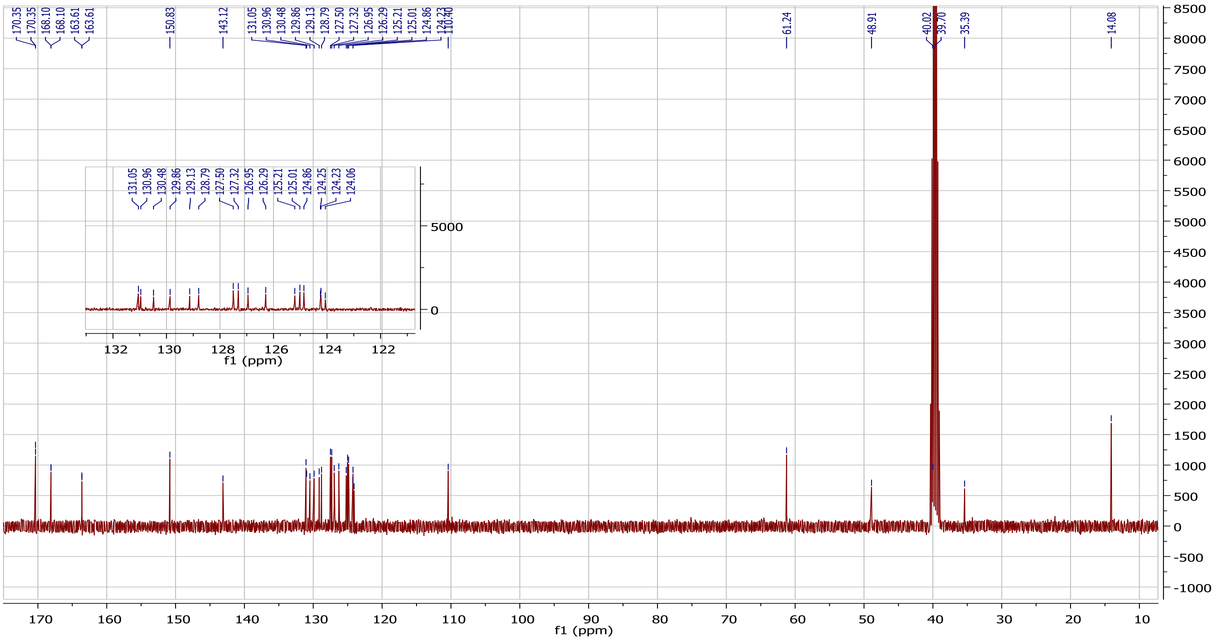


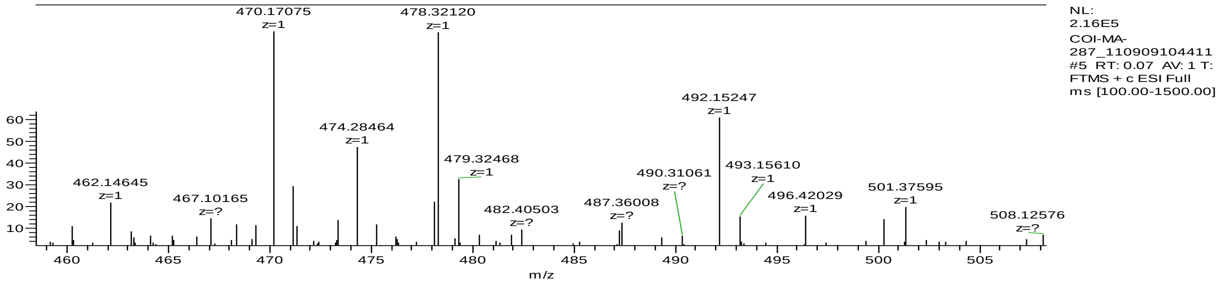


**Figure S2:** $^{1}$H NMR, $^{13}$C NMR (in DMSO-$d_6$) and HRMS of compound **4**.

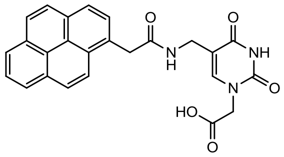


**5**

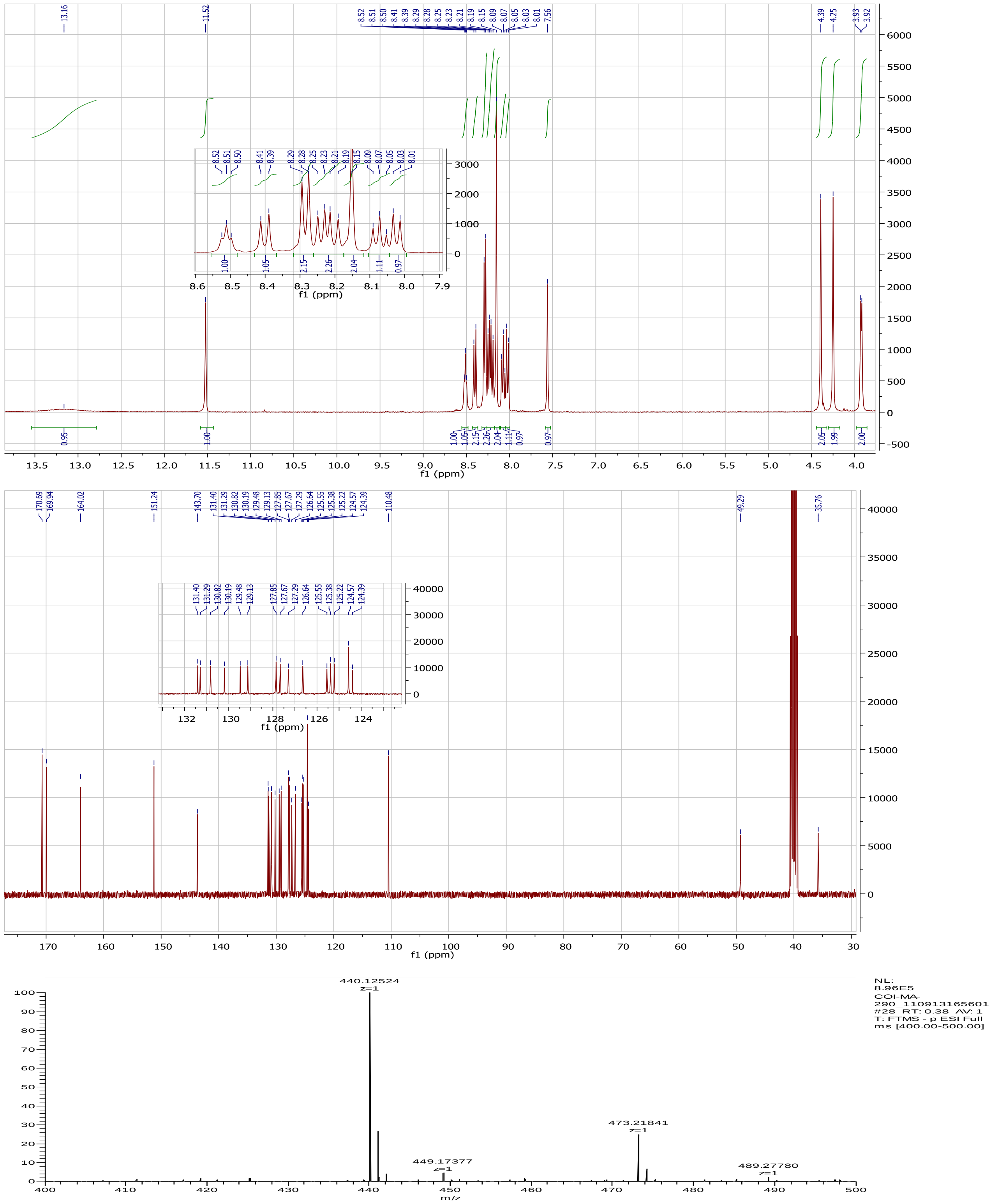


**Figure S3:** $^{1}$H NMR, $^{13}$C NMR (in DMSO-$d_6$) and HRMS of compound **5**.

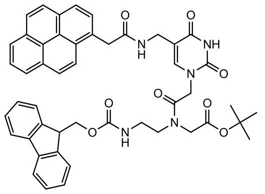

**6**

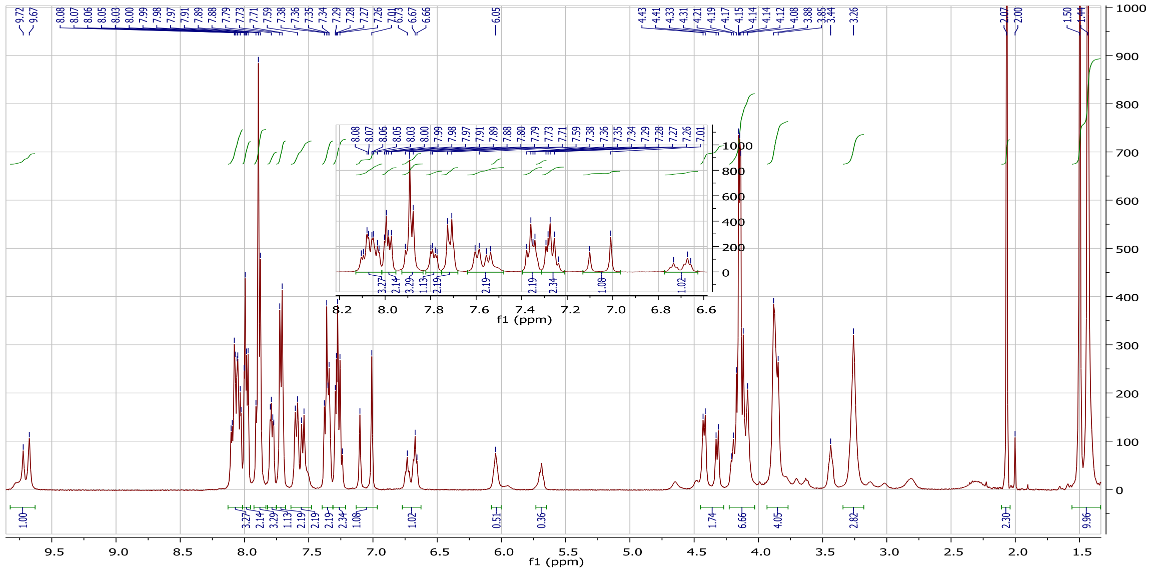


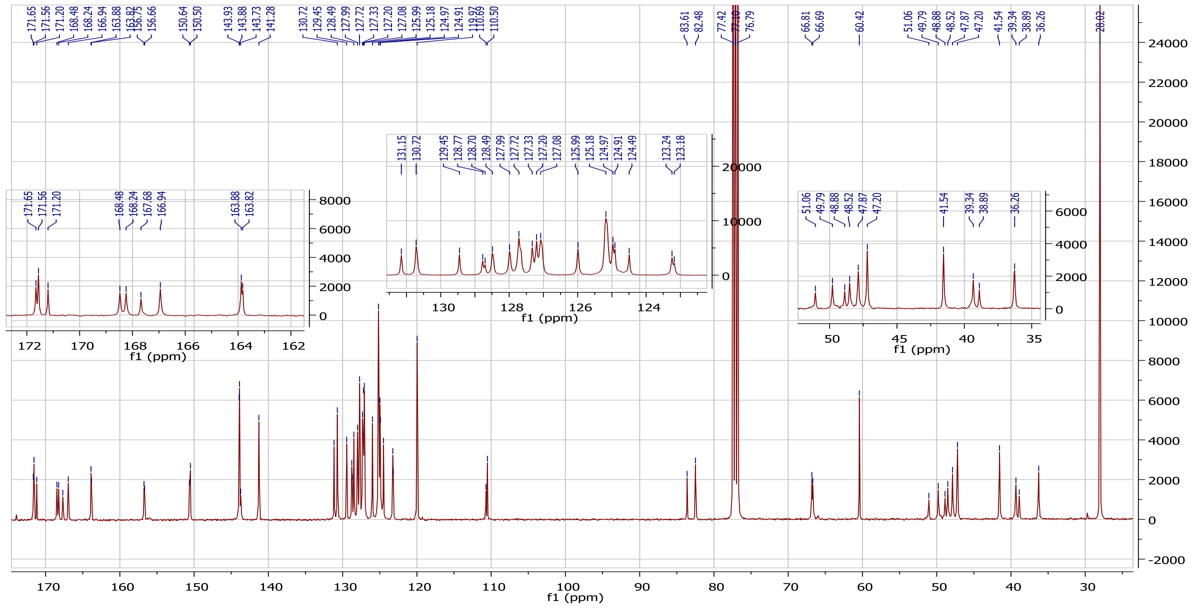


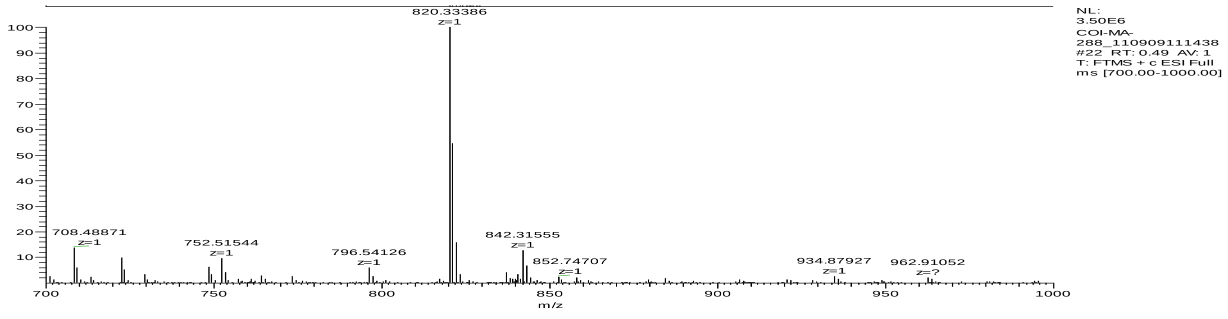


**Figure S4:** $^{1}$H NMR, $^{13}$C NMR (in DMSO-$d_6$) and HRMS of compound **6**.

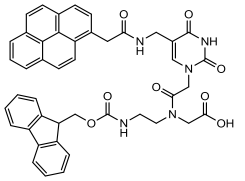

**1**

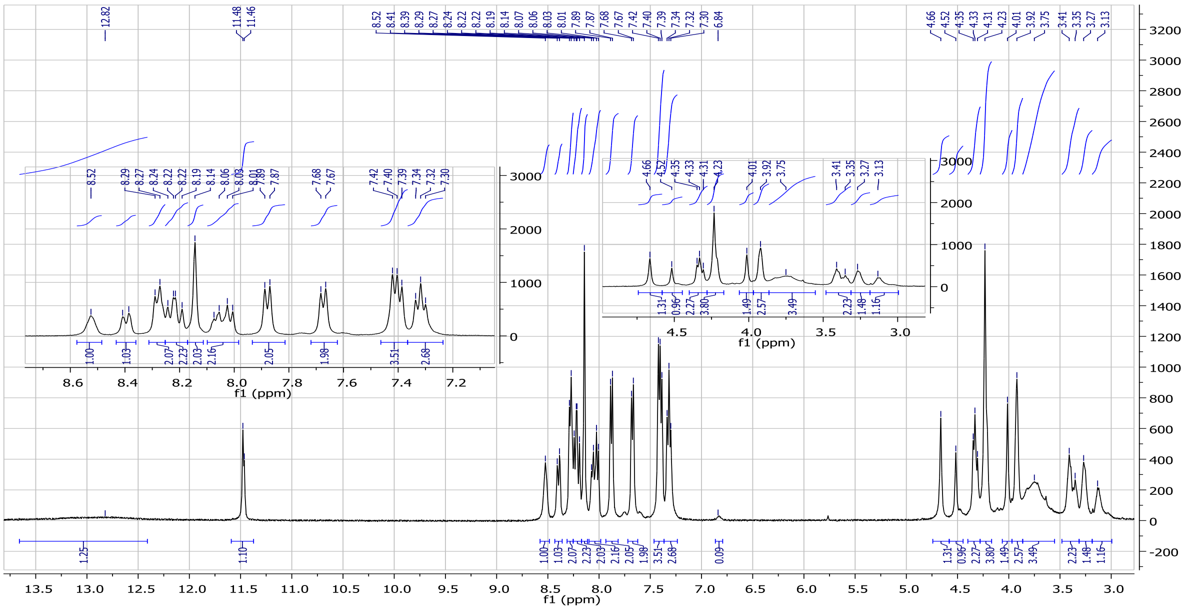

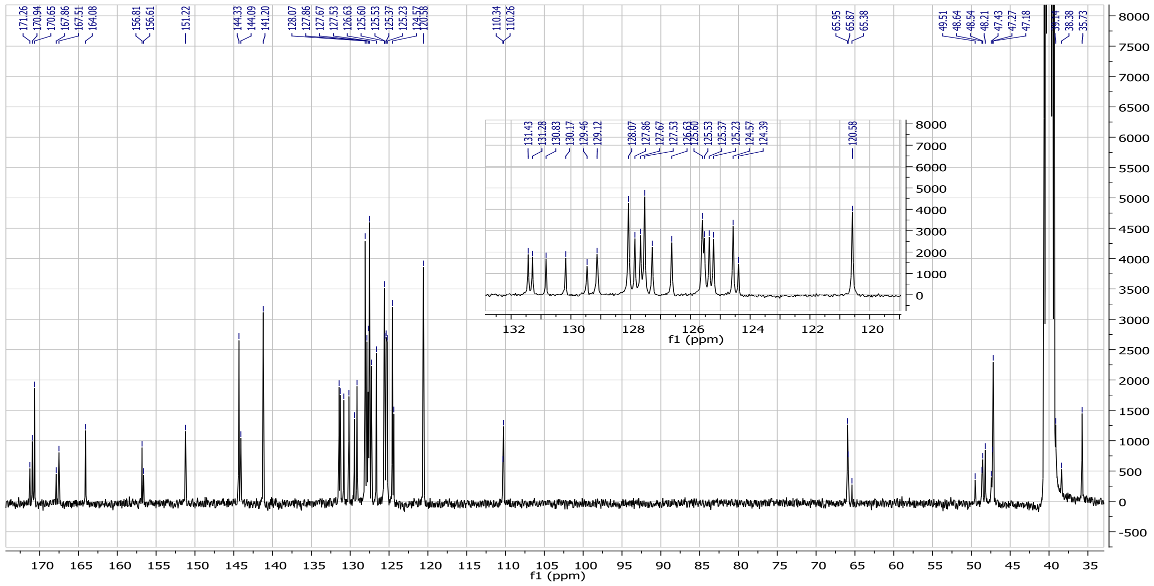

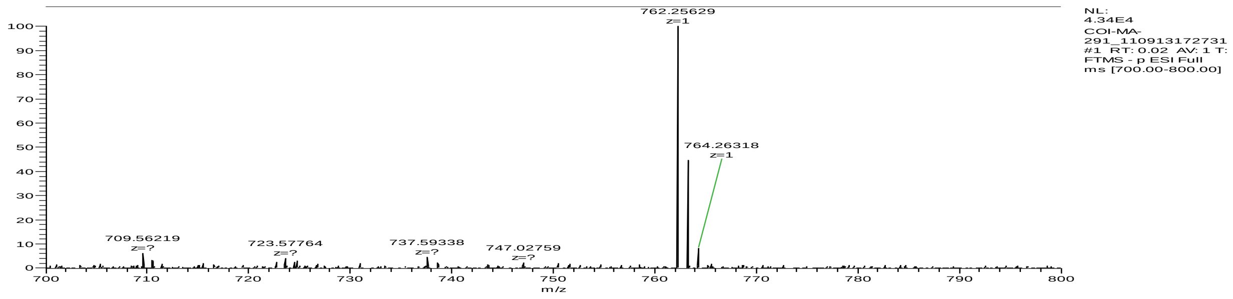


**Figure S5:** $^{1}$H NMR, $^{13}$C NMR (in DMSO-$d_6$) and HRMS of compound **1**.

# 3. Characterization of PNA 2–6

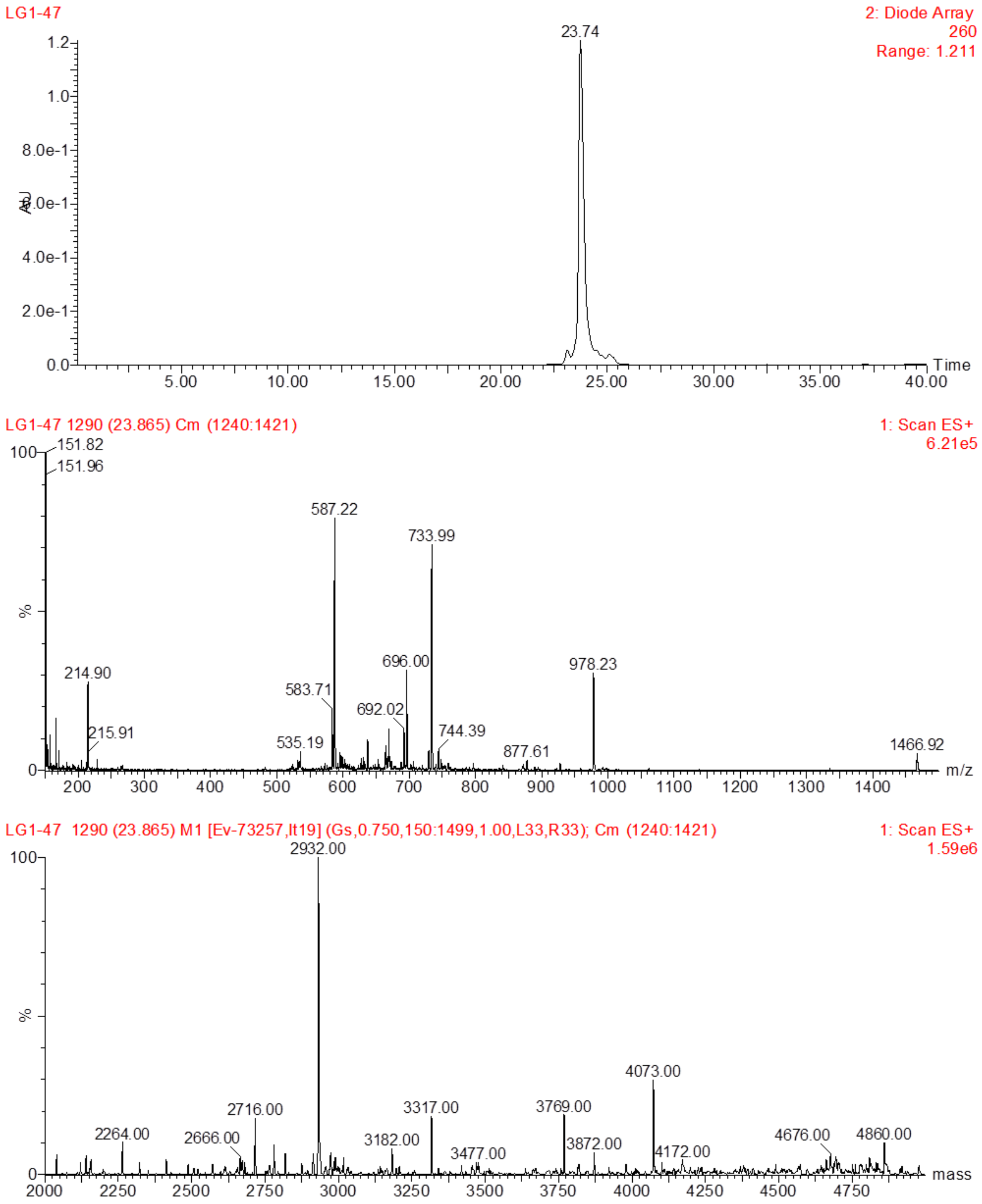


**Figure S6:** HPLC–MS of **PNA2**. Top to bottom: chromatographic profile, mass spectra of the major peak and reconstructed spectrum.

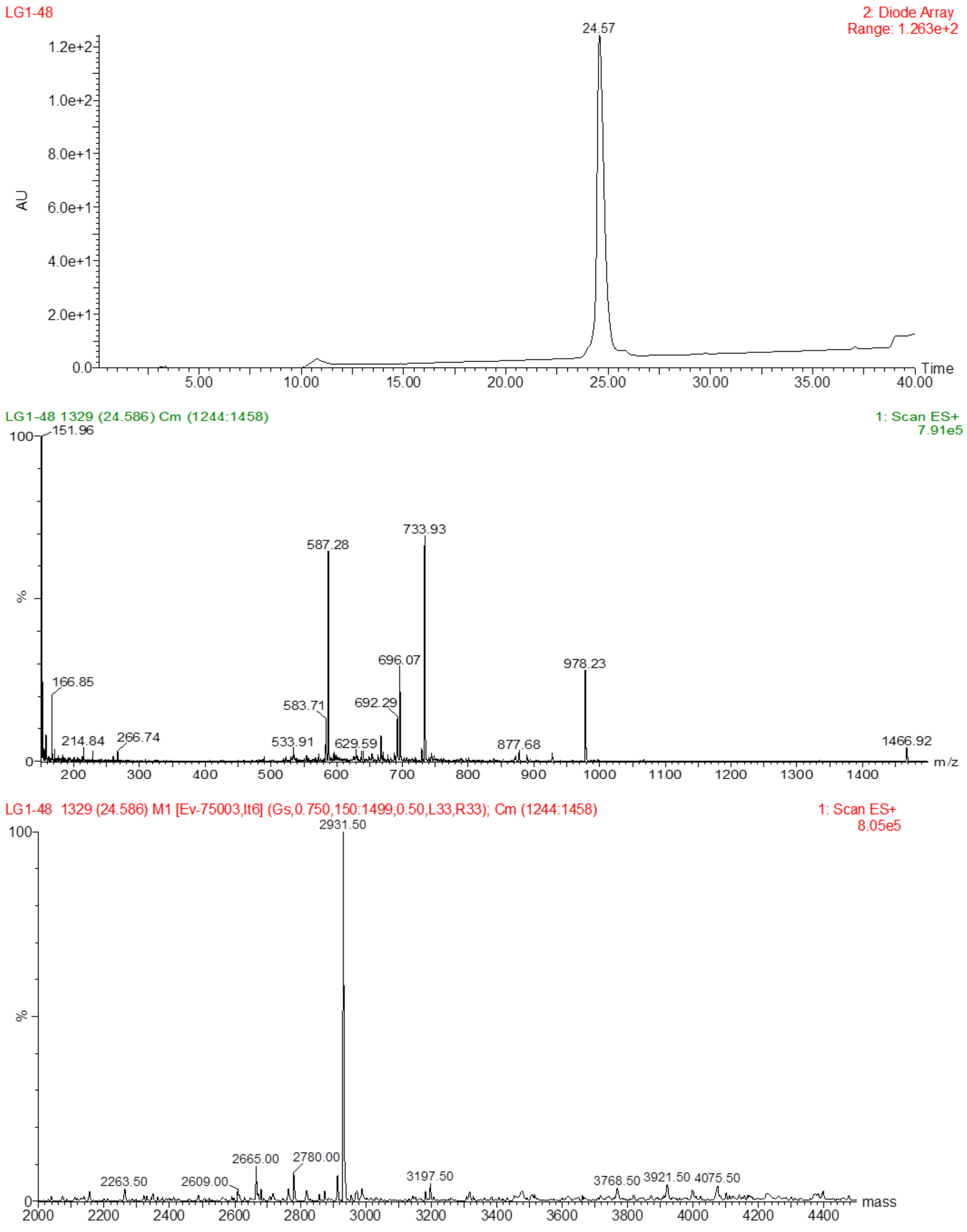


**Figure S7:** HPLC–MS of **PNA3**. Top to bottom: chromatographic profile, mass spectra of the major peak and reconstructed spectrum.

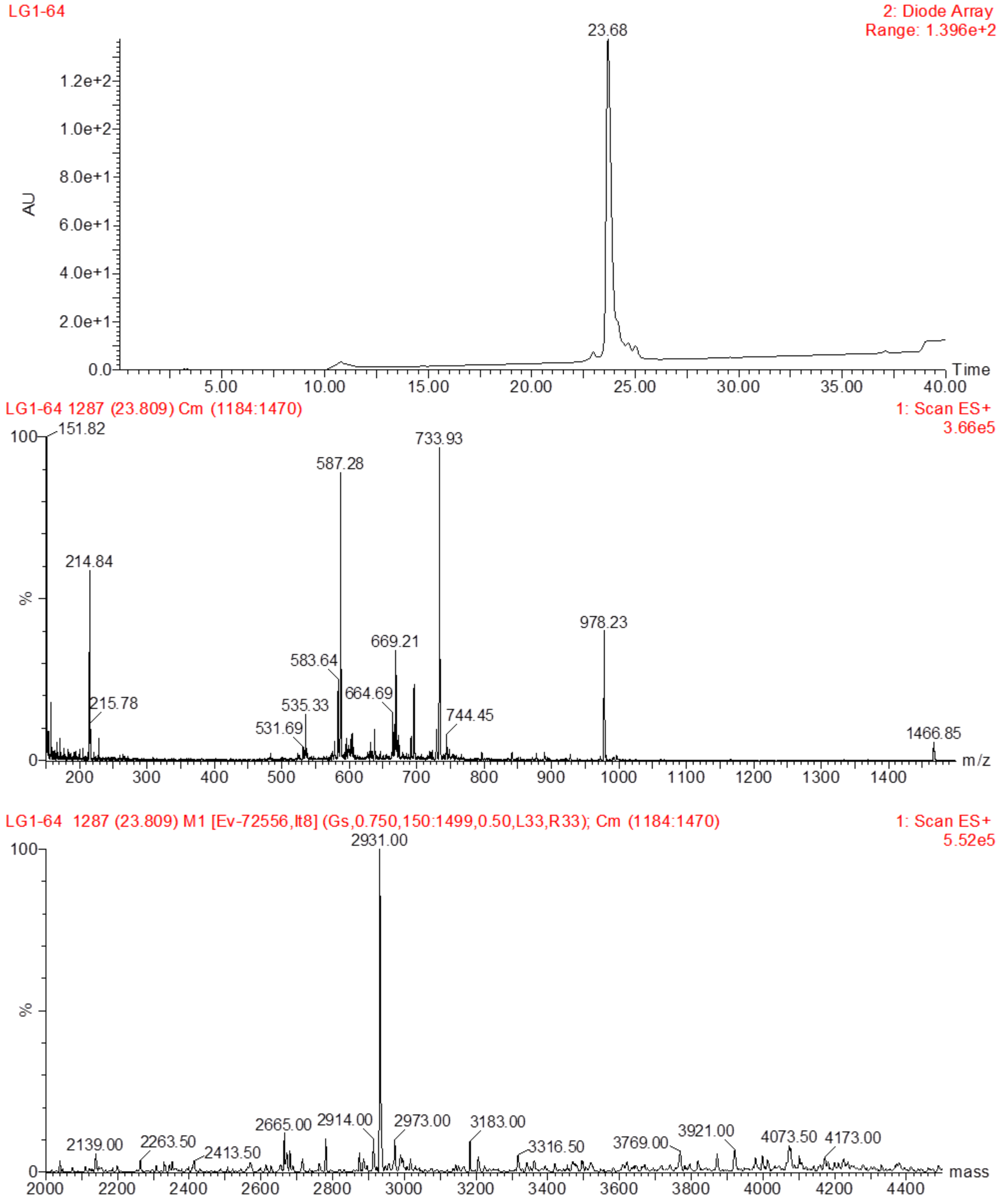


**Figure S8:** HPLC–MS of **PNA4**. Top to bottom: chromatographic profile, mass spectra of the major peak and reconstructed spectrum.

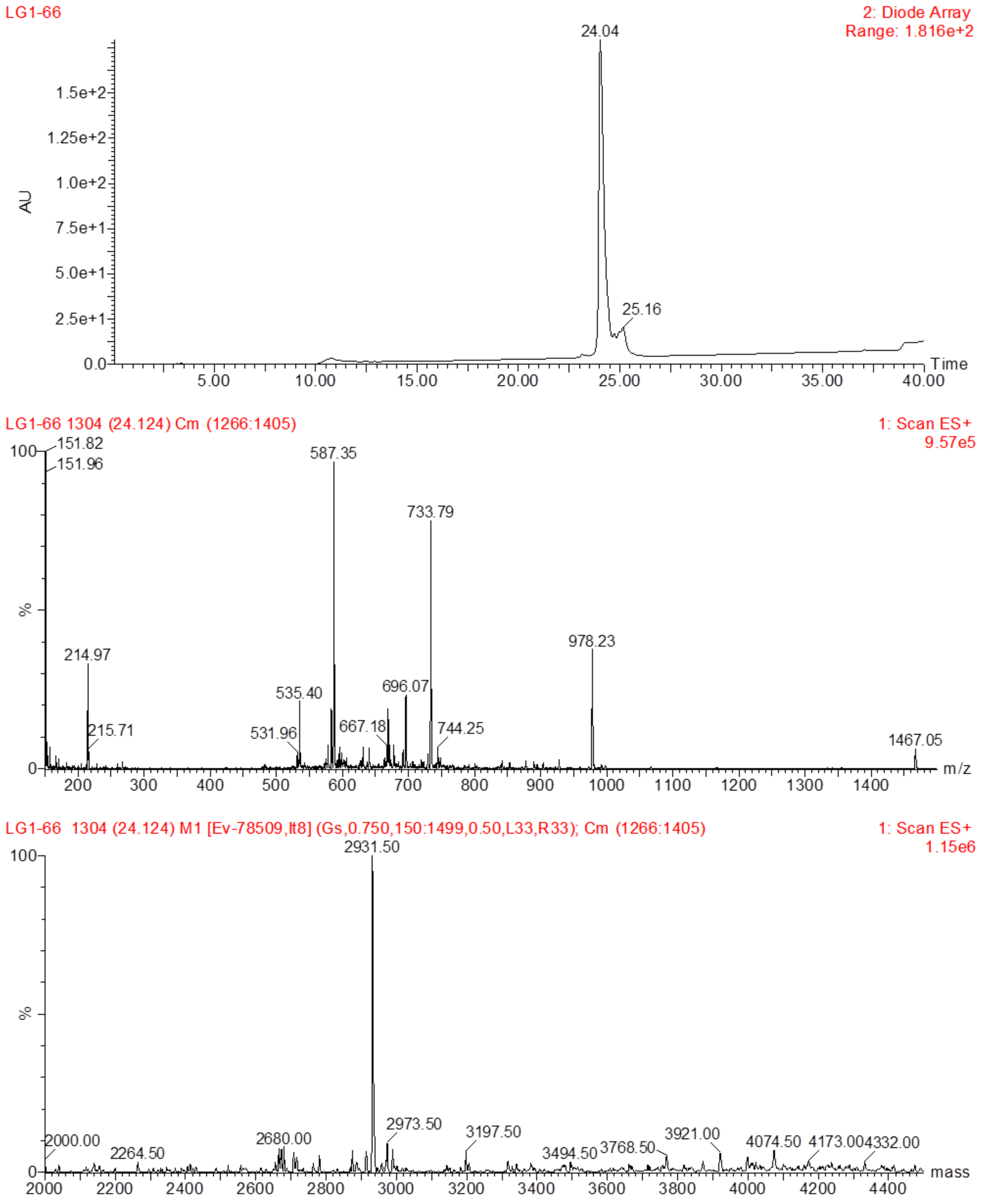


**Figure S9:** HPLC–MS of **PNA5**. Top to bottom: chromatographic profile, mass spectra of the major peak and reconstructed spectrum.

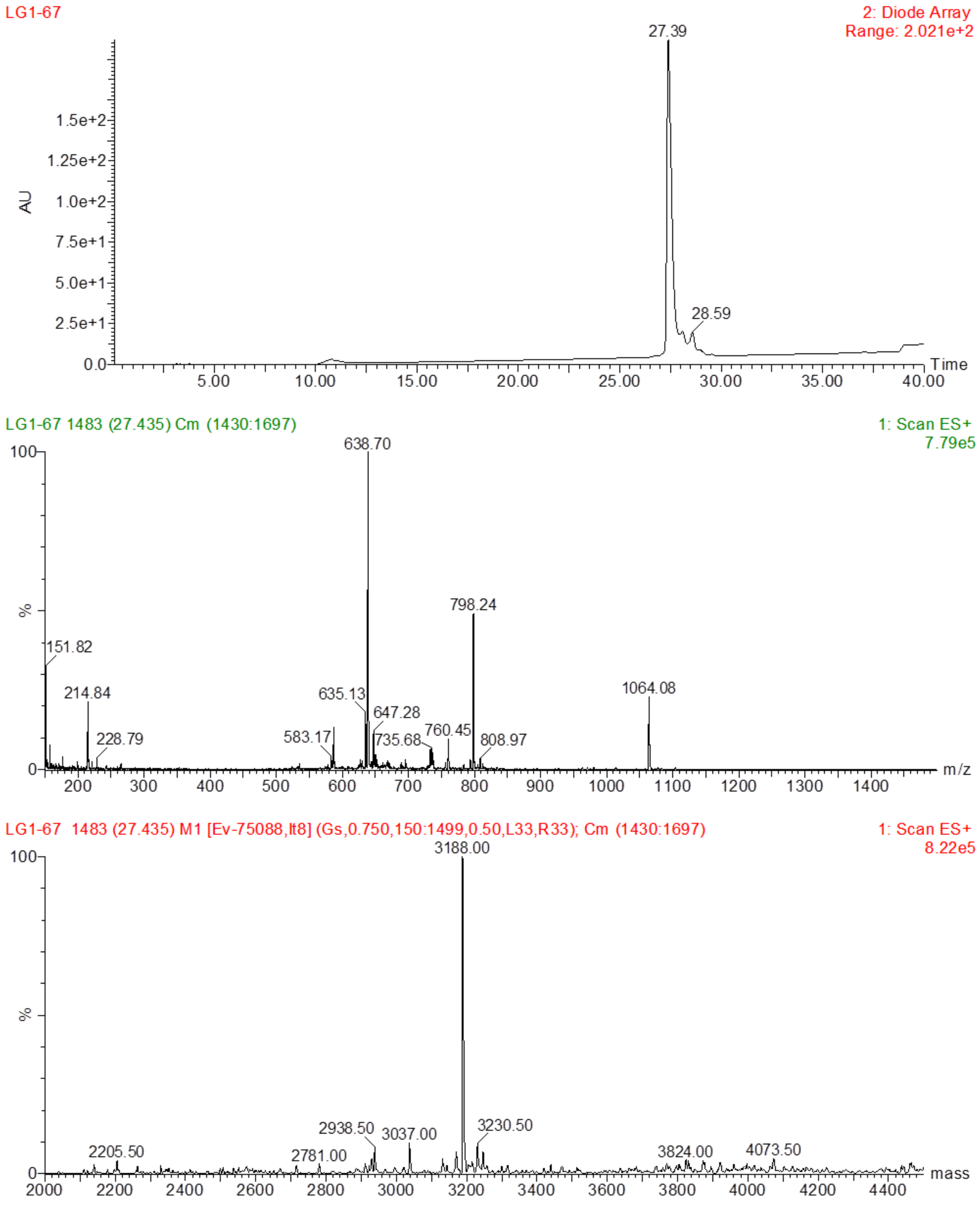


**Figure S10:** HPLC–MS of **PNA6**. Top to bottom: chromatographic profile, mass spectra of the major peak and reconstructed spectrum.

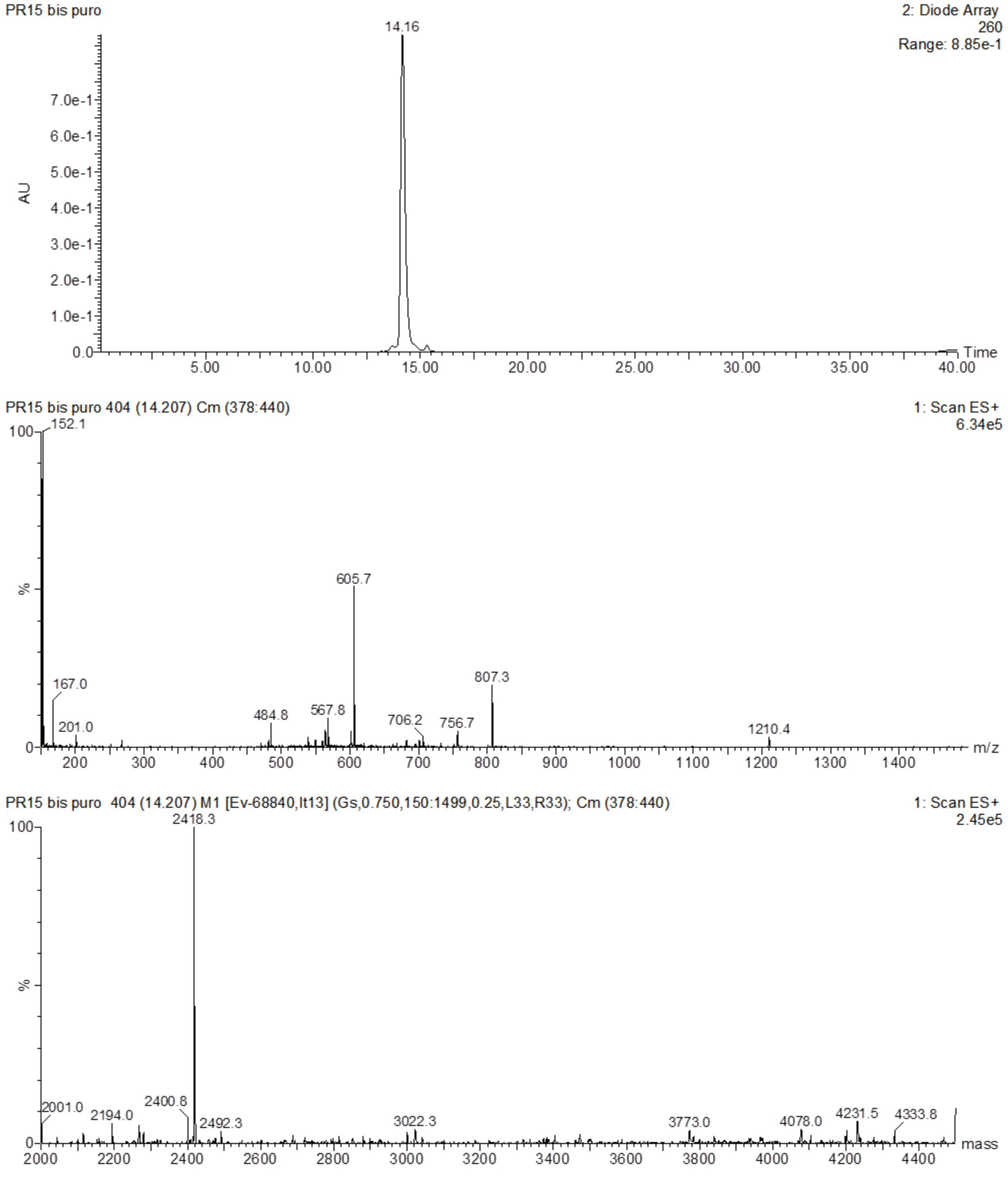


**Figure S11:** HPLC–MS of **PNA7**. Top to bottom: chromatographic profile, mass spectra of the major peak and reconstructed spectrum.

# 4. UV measurements

## 4.1 Calculation of the extinction coefficient for 5.

A stock solution of **5** was prepared in $H_2O$ (1.17 mg in 2.0 mL, 1.32 mM). From this solution an intermediate 1:10 dilution was prepared, which was used to prepare the final solutions with a 1:100, 1.5:100, 2:100, 3:100, 5:100, 7.5:100 dilution. Each solution was prepared in triplicate and the absorbance of the solutions were evaluated within the range of 200–500 nm at a scan speed of 100 nm/min.

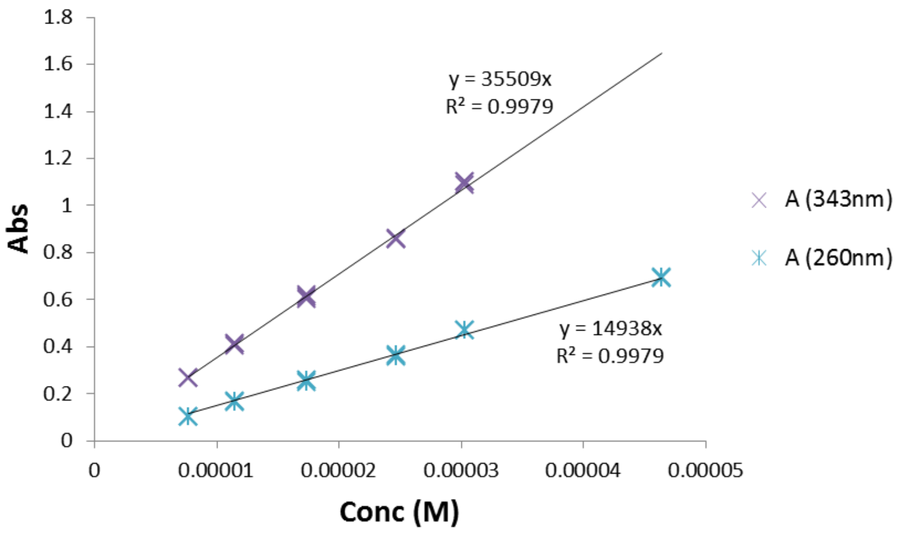


**Figure S12:** Linear regression for the evaluation of the extinction coefficient of **5** at 260 nm and 343 nm.

## 4.2 Variable temperature UV measurements.

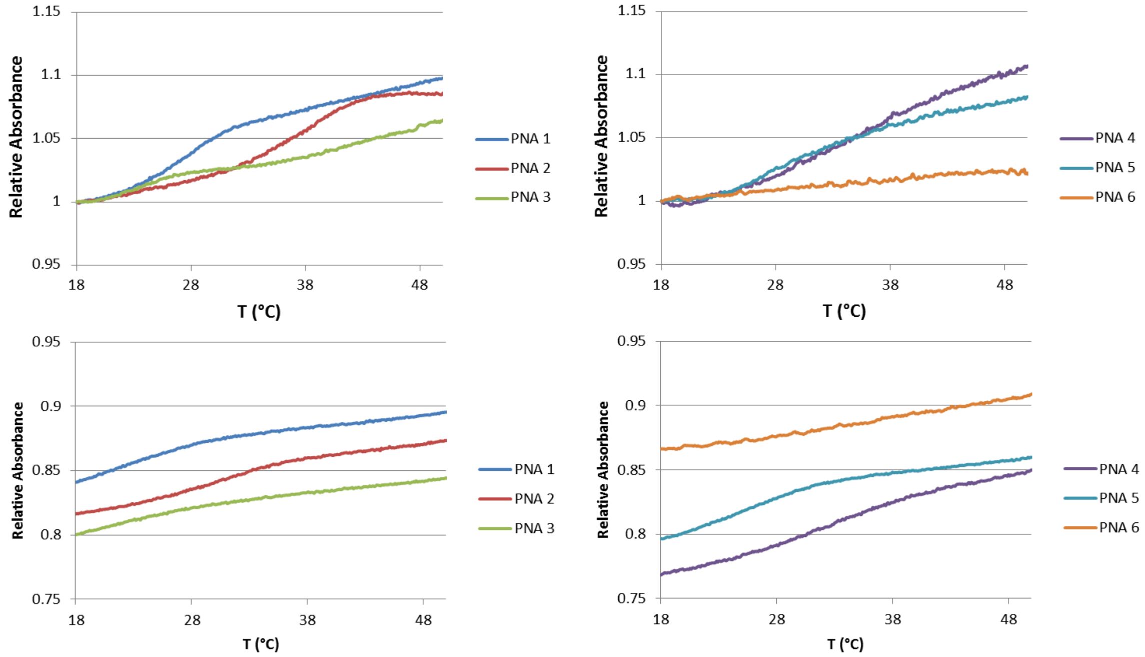


**Figure S13:** Relative absorbance at 260 nm as a function of temperature for PNA:**DNA1** complexes for melting (upper row) and annealing (lower row) processes. All measurements were done in PBS buffer, pH 7; concentration of each strand was 1 μM.

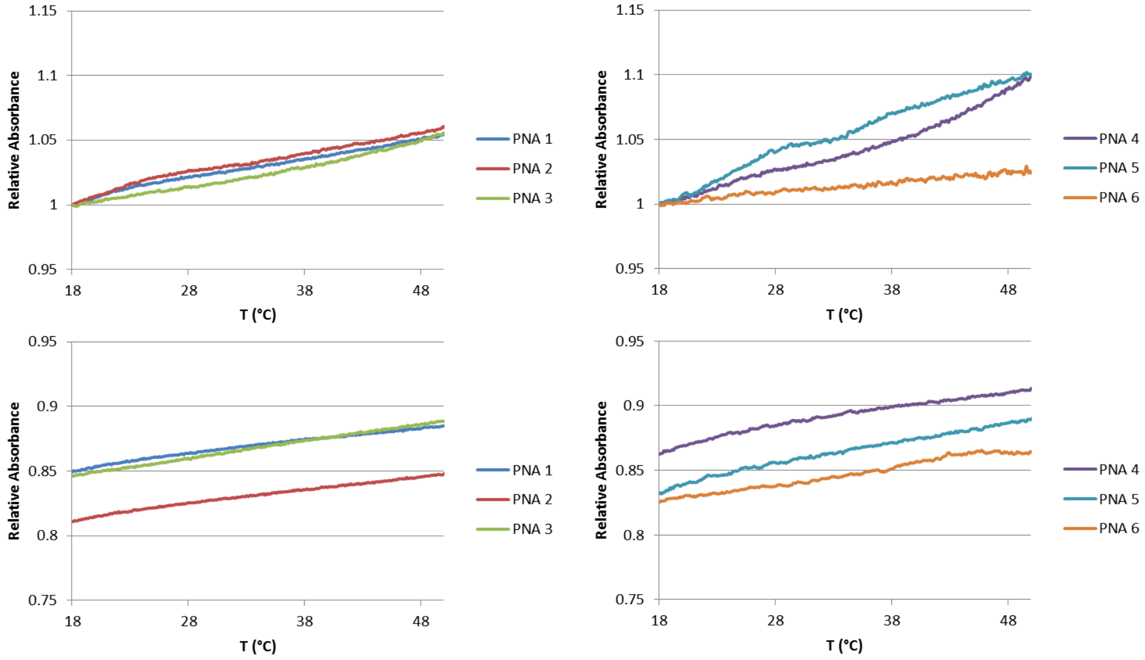


**Figure S14:** Relative absorbance at 260 nm as a function of temperature for PNA:**DNA2** complexes for melting (upper row) and annealing (lower row) processes. All measurements were done in PBS buffer, pH 7; concentration of each strand was 1 μM.

**Table S1:** UV melting temperatures of PNA:DNA complexes. All measurements were done in PBS at pH 7 and 1 μM strand concentration except for unmodified PNA measurements that were conducted at 5 μM strands concentration. $\Delta Tm$ refers to the difference between melting and annealing temperatures.

| PNA | With **DNA1** (°C) | | | With **DNA2** (°C) | | |
|---|---|---|---|---|---|---|
| | Melting | Annealing | $\Delta T_m$ | Melting | Annealing | $\Delta T_m$ |
| **PNA1** | 26 | 22 | 4 | 20 | < 18 | >2 |
| **PNA2** | 39 | 31 | 8 | 19 | < 18 | >1 |
| **PNA3** | 24 | < 18 | >6 | < 18 | < 18 | - |
| **PNA4** | 33 (broad) | 32 | 1 | 22 | < 18 | >4 |
| **PNA5** | 28 | 25 | 3 | 22 | < 18 | >4 |
| **PNA6** | No detectable | No detectable | – | No detectable | No detectable | – |

## 5. Fluorescence measurements

### 5.1 Fluorescence analysis at variable temperature.

Fluorescence spectra were recorded on a Perkin Elmer LS55 Luminescence Spectrometer equipped with a LAUDA ECOline RE104 temperature control system, exciting at 347 nm (slit: 5.0 nm), scanning from 370 nm to 550 nm, a scan speed of 200 nm/min was used and 3 accumulation for each spectrum. Samples were prepared as reported for UV measurements. All the samples were first incubated at 90 °C for 5 min, then slowly cooled to 10 °C. Fluorescence spectra were recorded every 5 °C allowing the sample to equilibrate 5 min to the new temperature before the new record. All measurements were compensated for lamp fluctuations by normalization using as reference a solution containing 20 nM 1-pyreneacetic acid in PBS. The results obtained are reported in Figure S15.

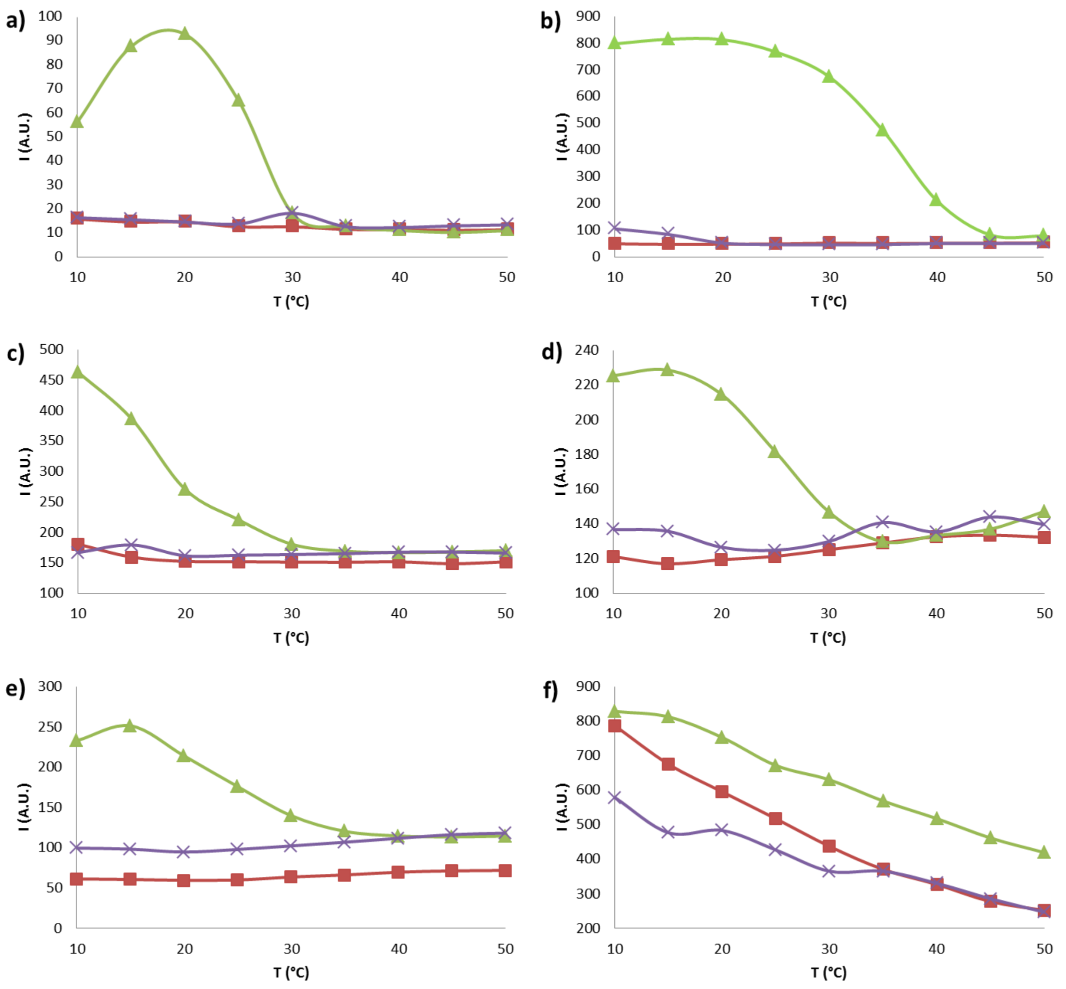


**Figure S15**: Fluorescence intensities of pyrene-modified PNA and their complexes as a function of temperature (excitation at 347 nm and emission at 470 nm); red squares are for ssPNA solutions, green triangles are for PNA:**DNA1** solutions and purple cross are for PNA:**DNA2** solutions; a) **PNA1**, b) **PNA2**, c) **PNA3**, d) **PNA4**, e) **PNA5**, f) **PNA6**. All measurements were done in PBS buffer at pH 7; concentration of each strand was 1 μM.

## 5.2 Fluorescence intensity of PNA2 relative to that of 1-pyreneacetic acid

From a 1 mM solution (2.6 mg in 100 ml) of 1-pyreneacetic acid in 10 mM NaOH, a 1 μM solution in PBS was prepared by dilution; the concentration of 1-pyreneacetic acid in this sample was checked by UV measurements at 347 nm; a 1 μM solution of **PNA2** was also prepared as described above. From these solutions samples of 200 nM concentration were prepared. Fluorescence emission spectra were recorded with an excitation wavelength of 347 nm (slit excitation: 5.0 nm), scanning from 370 nm to 550 nm (slit emission: 5.0 nm), a scan speed of 200 nm/min was used with 3 spectra accumulation for each solution. The spectra obtained are reported in Figure S15.

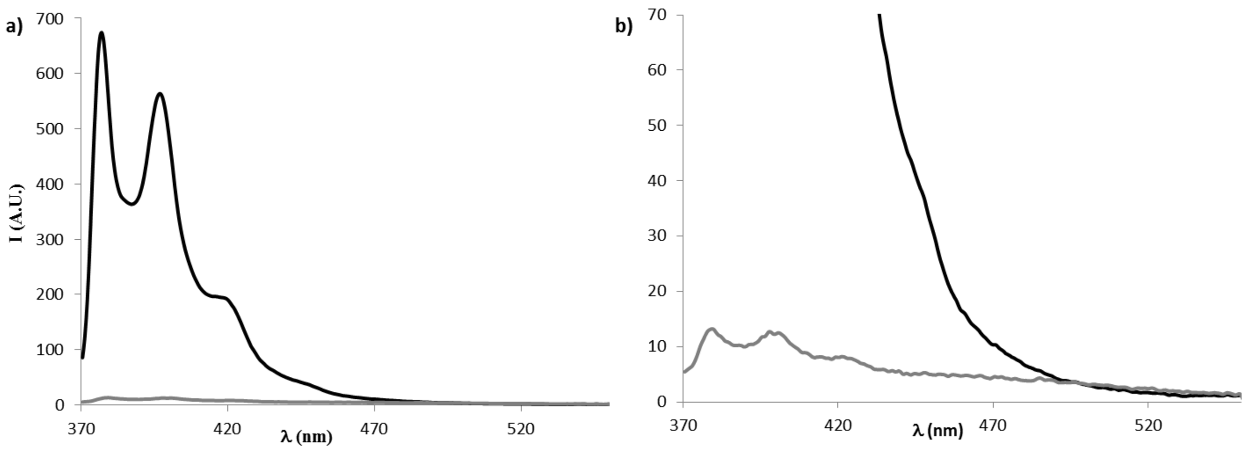


**Figure S16:** Fluorescence spectra (a) and 10× zoom (b) at 347 nm excitation, recorded at 20 °C. Grey lines: 200 nM **PNA2**; black lines: 200 nM 1-pyreneacetic acid. All measurement were done in PBS buffer, pH 7 at 20 °C.

The fluorescence quantum yield of the **PNA2** probe with excitation at 347 nm ($\phi_{PNA2}$) was compared with that of 1-pyreneacetic acid ($\phi_{pyr}$); the relative quantum yield ($\phi_{PNA2}/\phi_{pyr}$) at 20 °C was found to be 0.043. Since at the working conditions (0.2 μM concentration) the shapes of the emission spectra of 1-pyreneacetic and **PNA2** are the same, and the refractive indexes of both dilute solutions in the same buffer are the same, the following simplified formula was used:

$$\phi_{PNA2} / \phi_{pyr} = (I_{PNA2}/I_{Pyr}) \times [(1 - 10^{-A_{pyr}}) / (1 - 10^{-A_{PNA2}})]$$

where $I_{PNA2}$ and $I_{Pyr}$ refer to the integral of the emitted fluorescence of PNA2 and of 1-pyreneacetic acid, and $A_{pyr}$ and $A_{PNA2}$ are the corresponding absorbances at the excitation wavelength.

### 5.3 Fluorescence titration of PNA3

This titration was carried out as described for PNA 2 in the Experimental Part, but at 10°C in order to prevent complete dissociation of the complex (see Figure S15). The results are reported in Figure S17.

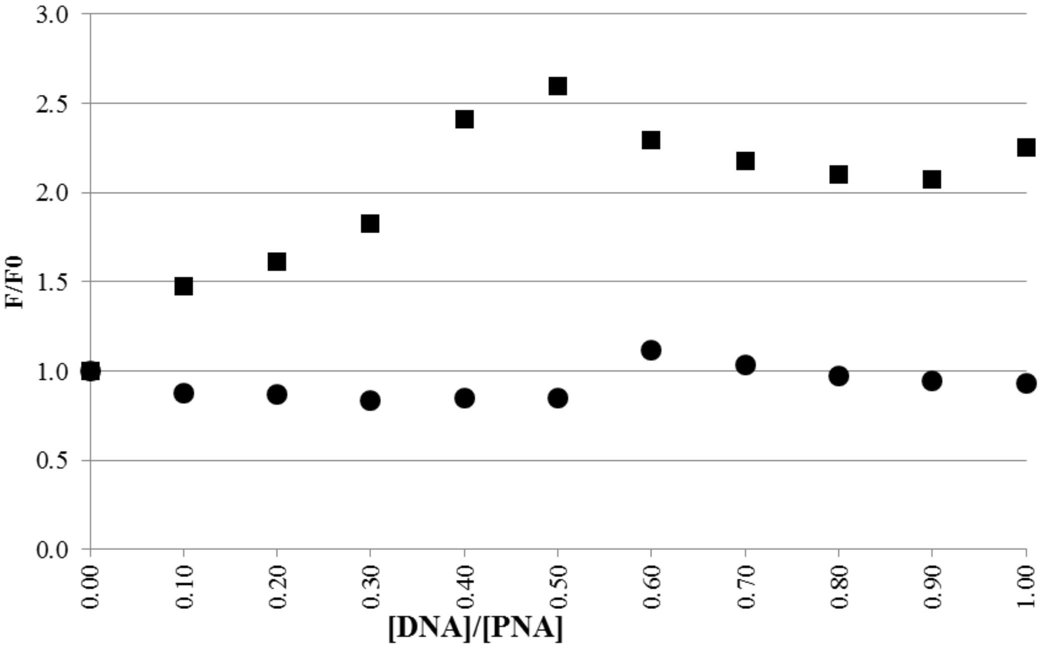


**Figure S17:** Fluorescence titration **PNA3** with **DNA1** (squares) or **DNA2** (circles). All measurements were done in PBS buffer, pH 7; concentration of each PNA strand was 1 μM. All data were normalized for the fluorescence intensity of the starting ssPNA solution. Conditions: $T$ = 10 °C, emission slit = 10 nm.

### 5.4 Determination of the limit of detection (LOD) for DNA1 using PNA2 as probe

From the stock solution described above single stranded **PNA2** solution (1 μM in PBS) and single stranded **DNA1** solution (4 μM in PBS) were prepared. From these, samples with different DNA concentrations (0, 20, 40, 60 and 80 nM, triplicate) were prepared. Fluorescence emission spectra were recorded with excitation wavelength of 347 nm (slit excitation: 5.0 nm), scanning from 370 nm to 550 nm (slit emission: 5.0 nm), a scan speed of 200 nm/min was used with 3 spectra accumulation for each solution, allowing each sample to incubate 10 minutes at 20 °C before analysis. From the data collected, using the 474 nm fluorescence intensity (I) as a function of **DNA1** concentration (nM), a linear regression was calculated: $I$ = 23±2 + 0.30±0.03·[**DNA1**] ($n$ = 12, $R^2$ = 0.911). A Mandel test was performed to confirm the significance of the linear model. From this linear regression the calculated LOD was 18.7 nM.

### 5.5 Base selectivity

Solutions of PNAs with all four base permutations were prepared at 1 µM PNA strand concentration as described above. All the samples were first incubated at 90 °C for 5 min, then slowly cooled to 10 °C. Fluorescence spectra were recorded every 5 °C allowing the sample to equilibrate 5 min to the new temperature before the new record. All measurements were compensated for lamp fluctuations by normalization using as reference a solution containing 20 nM 1-pyreneacetic acid in PBS. The results obtained are reported in Figure S18.

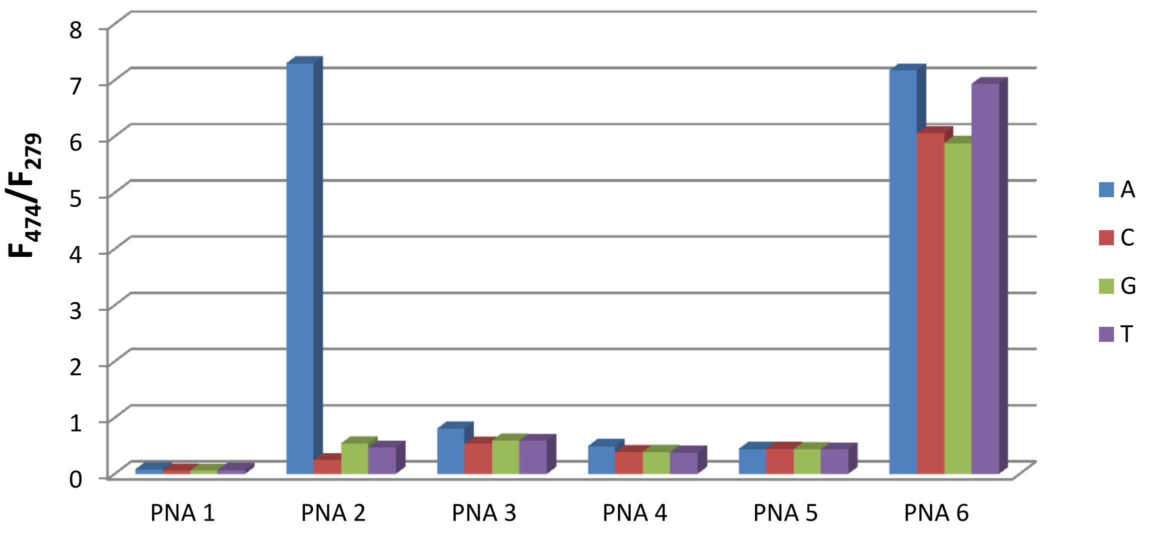


**Figure S18:** Normalized fluorescence 474 nm /379 nm ratio for PNA/DNA solution (5'-AGT GXA GGA-3', were X is the base reported in the legend). All measurements were done in PBS buffer, pH 7; concentration of each PNA strand was 1 µM and each DNA strand was 0.5 µM. All data were normalized for the fluorescence ratio of the **PNA2**/**DNA1** solution (X = adenine). Conditions: $T$ = 20 °C, emission slit = 10 nm, accumulation = 3.

## 6. Circular dichroism titration of PNA7

Circular dichroism spectra were recorded with a Jasco J715 spectropolarimeter and a PTC 348 temperature controller unit, collecting 3 scan each spectra. From a 100 µM stock solution a single stranded DNA solution (5 µM in PBS) was prepared, while the PNA stock solution was directly used for the titration. The DNA solution was first incubated at 15 °C then spectra were recorded after addition of portions of PNA (6.25 µL), allowing an equilibration time of 5 min. Solution concentration of DNA and PNA were checked by UV measurement to avoid dilution errors. The results are shown in Figure S19.

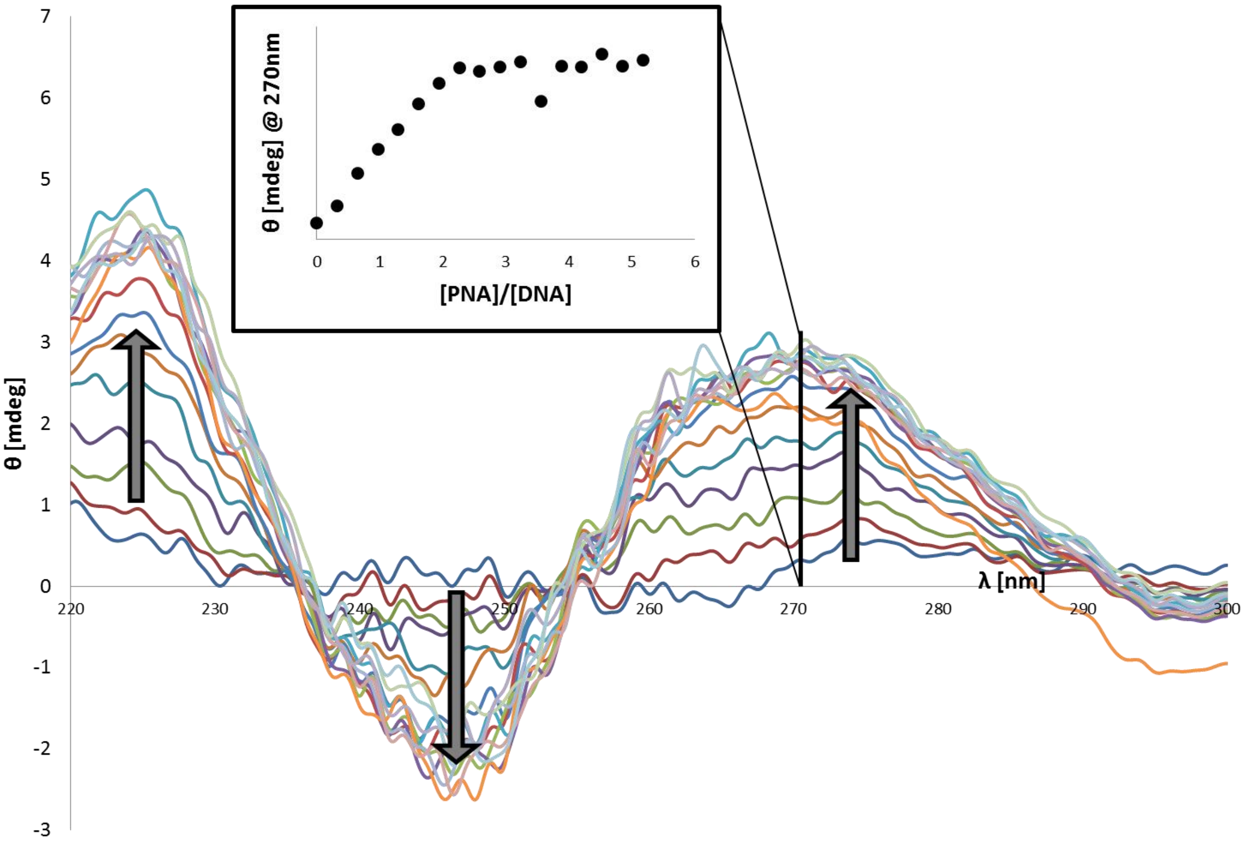


**Figure S19:** CD titration of **DNA1** with **PNA7** followed by circular dichroism. Arrows indicate signal variation after subsequent addition of PNA. Inside the frame: plot of the variation of the signal at 270 nm.

---

[1] Manicardi, A.; Accetta, A.; Tedeschi, T.; Sforza, S.; Marchelli, R.; Corradini, R. *Artif. DNA, PNA & XNA*, **2012**, *3*, 53-62.
